# Gene co-expression and histone modification signatures are associated with melanoma progression, epithelial-to-mesenchymal transition, and metastasis

**DOI:** 10.1186/s13148-020-00910-9

**Published:** 2020-08-24

**Authors:** Hátylas Azevedo, Guilherme Cavalcante Pessoa, Francisca Nathália de Luna Vitorino, Jérémie Nsengimana, Julia Newton-Bishop, Eduardo Moraes Reis, Júlia Pinheiro Chagas da Cunha, Miriam Galvonas Jasiulionis

**Affiliations:** 1 Division of Urology, Department of Surgery, Universidade Federal de São Paulo (UNIFESP), São Paulo, Brazil; 2 Department of Pharmacology, Universidade Federal de São Paulo (UNIFESP), Rua Pedro de Toledo 669 5 andar, Vila Clementino, São Paulo, SP 04039032 Brazil; 3 Laboratório de Ciclo Celular, Center of Toxins, Immune Response and Cell Signaling - CeTICS, Instituto Butantan, São Paulo, Brazil; 4 Institute of Medical Research at St James’s, University of Leeds School of Medicine, Leeds, UK; 5 Biostatistics Research Group, Population Health Sciences Institute, Newcastle University, Newcastle, United Kingdom; 6 Departamento de Bioquímica, Instituto de Química, Universidade de São Paulo, São Paulo, Brazil

**Keywords:** Melanoma, Co-expression network, Modules, Histones, Epithelial-to-mesenchymal transition, Metastasis

## Abstract

**Background:**

We have previously developed a murine cellular system that models the transformation from melanocytes to metastatic melanoma cells. This model was established by cycles of anchorage impediment of melanocytes and consists of four cell lines: differentiated melanocytes (melan-a), pre-malignant melanocytes (4C), malignant (4C11−), and metastasis-prone (4C11+) melanoma cells. Here, we searched for transcriptional and epigenetic signatures associated with melanoma progression and metastasis by performing a gene co-expression analysis of transcriptome data and a mass-spectrometry-based profiling of histone modifications in this model.

**Results:**

Eighteen modules of co-expressed genes were identified, and some of them were associated with melanoma progression, epithelial-to-mesenchymal transition (EMT), and metastasis. The genes in these modules participate in biological processes like focal adhesion, cell migration, extracellular matrix organization, endocytosis, cell cycle, DNA repair, protein ubiquitination, and autophagy. Modules and hub signatures related to EMT and metastasis (turquoise, green yellow, and yellow) were significantly enriched in genes associated to patient survival in two independent melanoma cohorts (TCGA and Leeds), suggesting they could be sources of novel prognostic biomarkers. Clusters of histone modifications were also linked to melanoma progression, EMT, and metastasis. Reduced levels of H4K5ac and H4K8ac marks were seen in the pre-malignant and tumorigenic cell lines, whereas the methylation patterns of H3K4, H3K56, and H4K20 were related to EMT. Moreover, the metastatic 4C11+ cell line showed higher H3K9me2 and H3K36me3 methylation, lower H3K18me1, H3K23me1, H3K79me2, and H3K36me2 marks and, in agreement, downregulation of the H3K36me2 methyltransferase Nsd1.

**Conclusions:**

We uncovered transcriptional and histone modification signatures that may be molecular events driving melanoma progression and metastasis, which can aid in the identification of novel prognostic genes and drug targets for treating the disease.

## Background

Melanoma is a skin tumor originated from melanocytes that exhibits high mortality when detected in advanced stages, due to its potential to invasion and metastasis. Patients with metastatic disease have shown poor survival rates of 10 to 20% of cases after 10 years from diagnosis [1]. Despite the hope that the current targeted therapies will improve the survival of patients with advanced disease, the long-term efficacy of these treatments may be limited by the frequent onset of drug resistance [2].

A better understanding of melanoma development and metastasis has been achieved by assessing the different stages of transformation from normal melanocytes to metastatic melanoma, according to the linear stepwise process proposed by Clark et al. [3]. The research in this area has revealed several molecular and biological processes underlying melanoma progression, such as genetic instability, deregulated proliferation, immunosuppression, angiogenesis, and epithelial-to-mesenchymal transition (EMT), followed by the overactivation of the MAPK, PI3K/PTEN/AKT, and MITF pathways [4].

The role of histone post-translational modifications (PTMs) has also been studied in melanoma progression [5]. Covalent modifications of histone amino acids are made by “writer” enzymes, such as methyltransferases and acetylases, and removed by “eraser” enzymes like demethylases or deacetylases [6]. These epigenetic changes shape the chromatin structure and result in activation or suppressive effects on transcription, cell cycle progression, and DNA damage/repair. For instance, the acetylation of lysine (K) residues neutralizes their positive charges and weakens the interaction of histones with the negatively charged DNA. This leads to a more open local chromatin conformation that increases the access to transcription factors [7]. In contrast, the stepwise methylation of histone lysines with 1–3 methyl groups can lead to enhancing or repressive transcriptional effects. While H3K4, H3K36, or H3K79 methylations are involved in transcriptional activation, the methylation of residues H3K27, H3K9, or H4K20 is linked to silenced chromatin [8].

To investigate molecular events that underlie melanoma progression, we have previously developed an in vitro model of melanoma by applying sequential detachment/re-adhesion cycles in non-tumorigenic murine melanocytes [9, 10]. The model comprises the following cell lines: melan-a (nonmalignant melanocytes), 4C (pre-malignant mesenchymal-like melanocytes), 4C11− (non-metastatic mesenchymal-like melanoma cells), and 4C11+ (metastatic melanoma cells). This system has allowed the molecular investigation of melanoma progression and metastasis by comparing the phenotypic differences of these cell lines. For instance, when subcutaneously inoculated to syngeneic mice, the 4C11− and 4C11+ cell lines grow as tumors, but only 4C11+ cells generate macrometastatic pulmonary foci [10, 11].

The transcriptional profiling of the four cell lines was previously performed using DNA microarray hybridization [11] and RNA sequencing [12]. This data has revealed that molecular changes related to the epigenetic machinery, immune response, and angiogenesis might play a role in melanoma progression [11] and underlie a reversible cellular phenotype switch between epithelial and mesenchymal-like states (Pessoa et al., submitted). In the current work, we have analyzed the transcriptome sequencing data using gene co-expression network modeling to identify key gene modules and provide further insights into the melanoma biology. Cancer signaling networks can be divided into modules of co-expressed genes that share common functions and are involved in tumor malignancy [13]. The genes in these modules are not equally connected: the most connected genes (hubs) have been reported as essential in cancer and are most likely to serve as biomarkers or drug targets [14]. Our analysis has identified hub genes and pointed to the involvement of epigenetic alterations in the transcriptional changes. Thus, we decided to systematically analyze the abundance dynamics of more than 240 histone PTM combinations in these cell lines to explore the connection between epigenetic modifications and gene co-expression patterns in melanoma progression and metastasis.

## Results

### Gene co-expression modules are related to tumor progression, EMT, and metastasis in a cellular model of melanoma progression

**Fig. 1:**
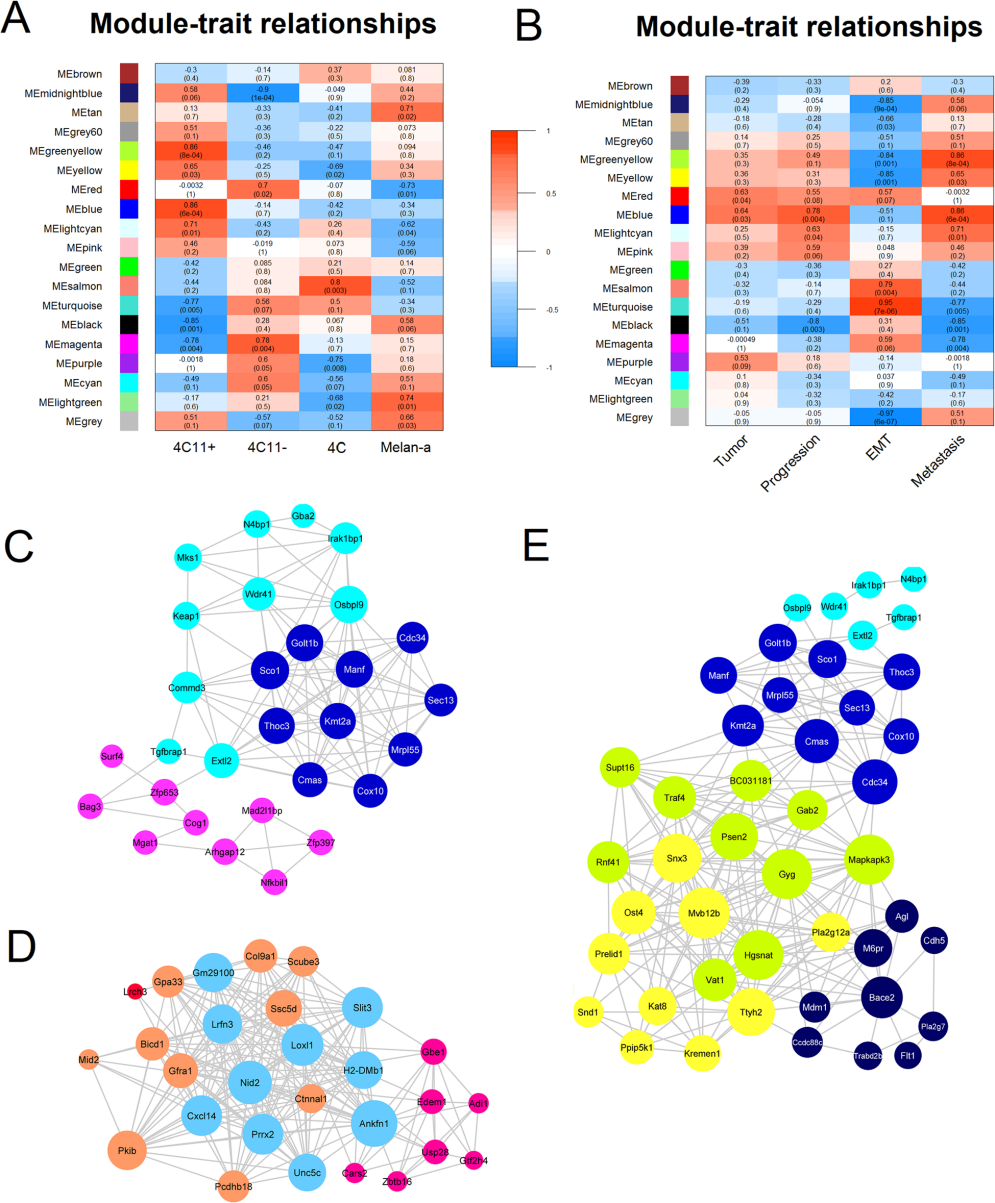
Co-expression modules and hubs associated with melanoma progression, EMT, and metastasis in the cellular model of melanoma progression. **a**, **b** Matrices containing the module-trait relationships for each cell line (**a**) or for tumor state, progression, EMT, and metastasis (**b**). Module names are shown on the y-axis, and correlation coefficients are displayed at the top of each row. Module-trait relationships were identified by calculating the Pearson correlation between module eigengenes and sample features classified using binary or sequential values. For assessing the relationship between tumor behavior and specific modules, the cell lines 4C11− and 4C11+ were assigned to the value 1 and the remaining cell lines were assigned to 0. For the EMT relationship, the mesenchymal-like 4C and 4C11− cell lines were assigned to 1 and the remaining epithelial cell lines were assigned to 0. For the metastasis relationship, only samples from the 4C11+ cell line were classified with the value 1. For tumor progression, the melan-a, 4C, 4C11−, and 4C11+ cell line samples were classified with the sequential numbers 1, 2, 3, or 4. Rows are colored based on the correlation sign of each module with the sample traits: red for positive (red) and negative (blue) correlation. The *p* values for each module are displayed at the bottom of each row within parentheses. Only modules with a correlation coefficient > 0.3 (absolute value) and a *p* value < 0.1 were considered significantly associated to a sample trait. **c–e** Subnetworks containing the co-expression interactions between the hubs from modules positively related to tumor progression (**c**), EMT (**d**), and metastasis (**e**). The hub genes from the modules that showed positive correlation with tumor progression (red, blue, light cyan, and pink), EMT (red, salmon, magenta, and turquoise), and metastasis (midnight blue, greenyellow, yellow, blue, and light cyan) were used to build the subnetworks related to these biological traits. Nodes were colored based on their module assignment, and node sizes were adjusted by degree centrality. Hubs were classified as the 10 top-ranked genes in each module based on intramodular connectivity (Kwithin). Networks were built using Cytoscape

An unsigned weighted gene co-expression network analysis (WGCNA) was performed to identify modules of co-expressed genes using the 5000 most variable genes across the cell lines. This analysis identified eighteen co-expressed gene modules in the murine cellular model of melanoma progression (Figure S2). The module sizes varied from 85 transcripts in the light cyan to 944 genes in the turquoise module, and transcripts not assigned to any module (*n* = 117) were allocated to the gray pseudo module. Significant relationships were found between the modules and specific cell lines (Fig. 1a) or between the modules and tumor progression, EMT, and metastasis (Fig. 1b). Table 1 shows the hub genes from the 14 modules that displayed significant relationship with the biological traits. Hubs were defined as the ten top-ranked genes in each module based on intramodular connectivity (Kwithin). Subnetworks were also built to show the connections between the hubs from these modules (Fig. 1c–e).

**Table 1:** Modules of co-expressed genes and their intramodular hubs

Module	Number of genes	Module-trait relationships (***p*** < 0.1 and correlation > ± 0.5)	Intramodular hubs (top 10 Kwithin values)
Turquoise	944	EMT and metastasis	*Slit3*, *Lrfn3*, *Gm29100*, *Ankfn1*, *Cxcl14*, *H2-DMb1*, *Unc5c*, *Loxl1*, *Nid2*, *Prrx2*
Blue	721	Tumor, progression, and metastasis	*Sec13*, *Cmas*, *Kmt2a*, *Sco1*, *Manf*, *Mrpl55*, *Golt1b*, *Thoc3*, *Cdc34*, *Cox10*
Yellow	509	EMT and metastasis	*Prelid1*, *Snx3*, *Ppip5k1*, *Kremen1*, *Ttyh2*, *Pla2g12a*, *Ost4*, *Kat8*, *Snd1*, *Mvb12b*
Red	295	Tumor, progression, and EMT	*Abhd10*, *Lrch3*, *Robo1*, *Ubl4a*, *Prim2*, *Hmces*, *4933423P22Rik*, *Slc2a4*, *Dlg1*, *Pdzd11*
Black	293	Progression and metastasis	*Prex1*, *Map2k4*, *Eepd1*, *Psma3*, *Fam131c*, *Cc2d1b*, *Prkx*, *Mbtps1*, *Tpk1*, *Neto2*
Magenta	149	EMT and metastasis	*Dnajc8*, *Cars2*, *Pank4*, *Edem1*, *Sap30*, *Mutyh*, *Usp28*, *Adi1*, *Zbtb16*, *Gbe1*
Purple	130	Tumor	*Gpat4*, *Pithd1*, *Pex14*, *A430005L14Rik*, *Dgat2*, *Mtfr1l*, *Psmb2*, *Rps6*, *Brf2*, *Coprs*
Green yellow	122	EMT and metastasis	*Gyg*, *Hgsnat*, *Rnf41*, *Psen2*, *Mapkapk3*, *BC031181*, *Vat1*, *Gab2*, *Traf4*, *Supt16*
Tan	119	EMT	*Kif4*, *Sra1*, *Tmem38a*, *Aco2*, *Coq10b*, *Klhl15*, *Mapre1*, *Mark4*, *1500011B03Rik*, *Mttp*
Salmon	118	EMT	*Pcdhb18*, *Scube3*, *Pkib*, *Mid2*, *Gpa33*, *Bicd1*, *Gfra1*, *Col9a1*, *Ssc5d*, *Ctnnal1*
Midnight blue	109	EMT and metastasis	*Trabd2b*, *Ccdc88c*, *Mdm1*, *Bace2*, *M6pr*, *Pla2g7*, *Ube2t*, *Cdh5*, *Agl*, *Flt1*
Light cyan	85	Progression and metastasis	*N4bp1*, *Mks1*, *Irak1bp1*, *Wdr41*, *Keap1*, *Tgfbrap1*, *Extl2*, *Commd3*, *Osbpl9*, *Gba2*
Pink	178	Progression	*Arhgap12*, *Mad2l1bp*, *Nfkbil1*, *Zfp397*, *Plpp1*, *Mgat1*, *Bag3*, *Zfp653*, *Surf4*, *Cog1*
Brown	519	No relationship	Yme1l1, Psmc6, Ranbp2, Nol8, Fam208b, Ap4e1, Prpf4b, Uba6, Ckap5, Bod1l

Hubs were defined as the top 10 ranked genes in each module based on Kwithin values. Module-trait relationships were considered to be significant if the *p* value < 0.1 and the correlation ≥ 0.5

The red, blue, light cyan, and pink modules were positively correlated with tumor progression (Fig. 1b). The genes in these modules participate in DNA damage response, DNA replication, mitochondrial translation, ubiquitination and proteasome degradation, cell migration, cytoskeleton organization, cell cycle, HATs acetylate histones, endocytosis, as well as in the MAPK, TNF-alpha, NF-kB, Hippo, Delta-Notch, Ras and Wnt signaling pathways (Table 2). In the subnetwork related to tumor progression (Fig. 1c), the hubs from the blue module are the most connected, such as the genes *Cmas*, *Kmt2a*, *Golt1b*, *Cdc34*, *Manf*, *Sco1*, and *Thoc3*, followed by hubs from the light cyan (Extl2, Commd3, Wdr41, Keap1, Osbpl9) and pink modules.

**Table 2:** Histone marks and biological terms enriched by the genes in each module

Module	Histone marks	Biological terms	Database
**Turquoise**	H3K27me3	Axon guidance	GO biological process
		Regulation of MAP kinase activity	
		Extracellular matrix organization	Reactome
		Delta-Notch signaling pathway	KEGG
		Glycosaminoglycan biosynthesis	
		Focal Adhesion-PI3K-Akt-mTOR-signaling pathway	WikiPathways
		Alpha6-Beta4 Integrin signaling pathway	
		TGF Beta signaling pathway	
**Blue**	H3K79me3, H3K79me2, H3K27ac, H3K4me3, H3K36me3, H4K20me1	Cellular response to DNA damage stimulus	GO biological process
		Mitochondrial translation	
		Ubiquitination and proteasome degradation	Reactome
		Negative regulation of MAPK pathway	
		Proteasome degradation	WikiPathways
		TNF-alpha NF-kB signaling pathway	
		Protein processing in endoplasmic reticulum	KEGG
**Yellow**	H3K4me3, H3K36me3	Mitochondrion organization	GO biological process
		Hippo signaling pathway	KEGG
		Endocytosis	
		Lysosome	
**Red**	-	Regulation of cell migration	GO biological process
		Cell cycle	Reactome
		Axon guidance	KEGG
		Hippo signaling pathway	KEGG
		DNA replication	KEGG
		G1 to S cell cycle control	WikiPathways
		ESC pluripotency pathways	WikiPathways
**Black**	H3K27ac, H3K79me2	Activation of JUN kinase activity	GO biological process
		Positive regulation of apoptotic process	
		Regulation of small GTPase signal transduction	
		TNF signaling pathway	KEGG
		Regulation of actin cytoskeleton	KEGG
		Protein processing in endoplasmic reticulum	KEGG
		Tight junction	KEGG
		Regulation of actin cytoskeleton	WikiPathways
		Wnt signaling pathway and pluripotency	WikiPathways
**Magenta**	H3K27ac, H3K4me3	mRNA splicing	GO biological process
		Double-strand break repair	
		DNA replication	WikiPathways
		Nucleotide excision repair	KEGG
		Spliceosome	KEGG
**Purple**	H3K79me2, H3K36me3	Protein ubiquitination	GO biological process
		RNA processing	
		Insulin signaling pathway	KEGG
		Endocytosis	KEGG
		Ribosome	KEGG
		Delta-Notch signaling pathway	WikiPathways
		Wnt signaling pathway	WikiPathways
**Greenyellow**	H3K79me2	Triglyceride biosynthesis	Reactome
		Autophagosome organization	GO biological process
		Positive regulation of cytoskeleton organization	
		Ras protein signal transduction	
**Tan**	-	Base excision repair	KEGG
		Signaling by Rho GTPases	Reactome
		DNA repair	Reactome
		Regulation of cell cycle	GO biological process
		Protein ubiquitination	
**Salmon**	-	Regulation of toll-like receptor signaling pathway	WikiPathways
		Focal adhesion	KEGG
		ECM-receptor interaction	KEGG
		p75 NTR receptor-mediated signaling	Reactome
		Cell cycle	KEGG
**Midnight blue**	-	Focal adhesion	KEGG
		Insulin receptor signaling cascade	Reactome
		Signaling events mediated by VEGFR	NCI-NATURE
		Positive regulation of MAP kinase activity	GO biological process
		Neutrophile degranulation	
**Light cyan**	H3K79me2	HATs acetylate histones	Reactome
		Regulation of mitotic cell cycle	GO biological process
**Pink**	H3K27ac	Regulation of mRNA stability	GO biological process
		Proteasomal protein catabolic process	GO biological process
**Brown**	H3K79me2, H3K27ac, HeK4me3, H3ac, H3K9ac, H3K36me3	mRNA processing	WikiPathways
		Homologous recombination	KEGG
		Cell cycle	Reactome
		Cellular response to DNA damage stimulus	GO biological process

The functions, pathways, and histone marks were considered to be significantly enriched if their adjusted *p* values were less than 0.05

In the blue module, *Cdc34* codes for a ubiquitin-conjugating enzyme that regulates cell cycle progression, while *Cmas* encodes a key enzyme (cytidine monophosphate N-acetylneuraminic acid synthetase) in the sialylation pathway that enhances invasion of breast cancer cells [15]. In the light cyan module, *Extl2* (Exostosin-like 2) codes for a glycosyltransferase from the exostosin family that modifies the heparin sulfate structure and is involved with breast cancer cell adhesion and invasion [16]. In addition, the hub gene *Commd3* (COMM domain-containing 3) was shown to regulate c-myc transcription and prostate tumor growth in mice [17].

The red, salmon, turquoise, and magenta modules were positively correlated with EMT (Fig. 1b). The genes in these modules participate in functions such as axon guidance, focal adhesion, cell migration, MAP kinase activity, extracellular matrix organization, delta-Notch pathway, hippo signaling pathway, endocytosis, cell cycle, DNA replication and repair, spliceosome, and protein ubiquitination. In the subnetwork related to EMT (Fig. 1d), hubs from the turquoise module are the most connected (depicted as circles with larger node size), such as the genes *Nid2*, *Loxl1*, *Slit3*, *Lrfn3*, *Cxcl14*, *Prrx2*, and *Ankfn1* followed by nodes from the salmon, magenta, and red modules.

In the turquoise module, the hub genes *Cxcl14* and *Slit3* are known to inhibit EMT by suppressing respectively the NF-κB and Robo signaling pathways; they were shown to reduce cell migration in melanoma [18, 19]. *Loxl1* codes for an enzyme that oxidizes extracellular matrix proteins, remodels the tumor microenvironment, and is involved in EMT and metastasis [20]. In the salmon module, the hub *Scube3* is a TGF-β receptor ligand that induces EMT in lung cancer [21], whereas *Ctnnal1* (catenin alpha-like 1) inhibits EMT in bronchial epithelial cells by reducing TGF-β levels [22]. In addition, the hubs *Nid2* (nidogen-2) and *Lrfn3* (leucine-rich repeat and fibronectin type III domain-containing 3) from the turquoise module and the hubs *Pcdhb18* (protocadherin beta 18 pseudogene) and *Col9a1* (collagen type IX alpha 1 chain) from the salmon modules are associated with extracellular matrix and cell adhesion-related functions.

**Fig. 2:**
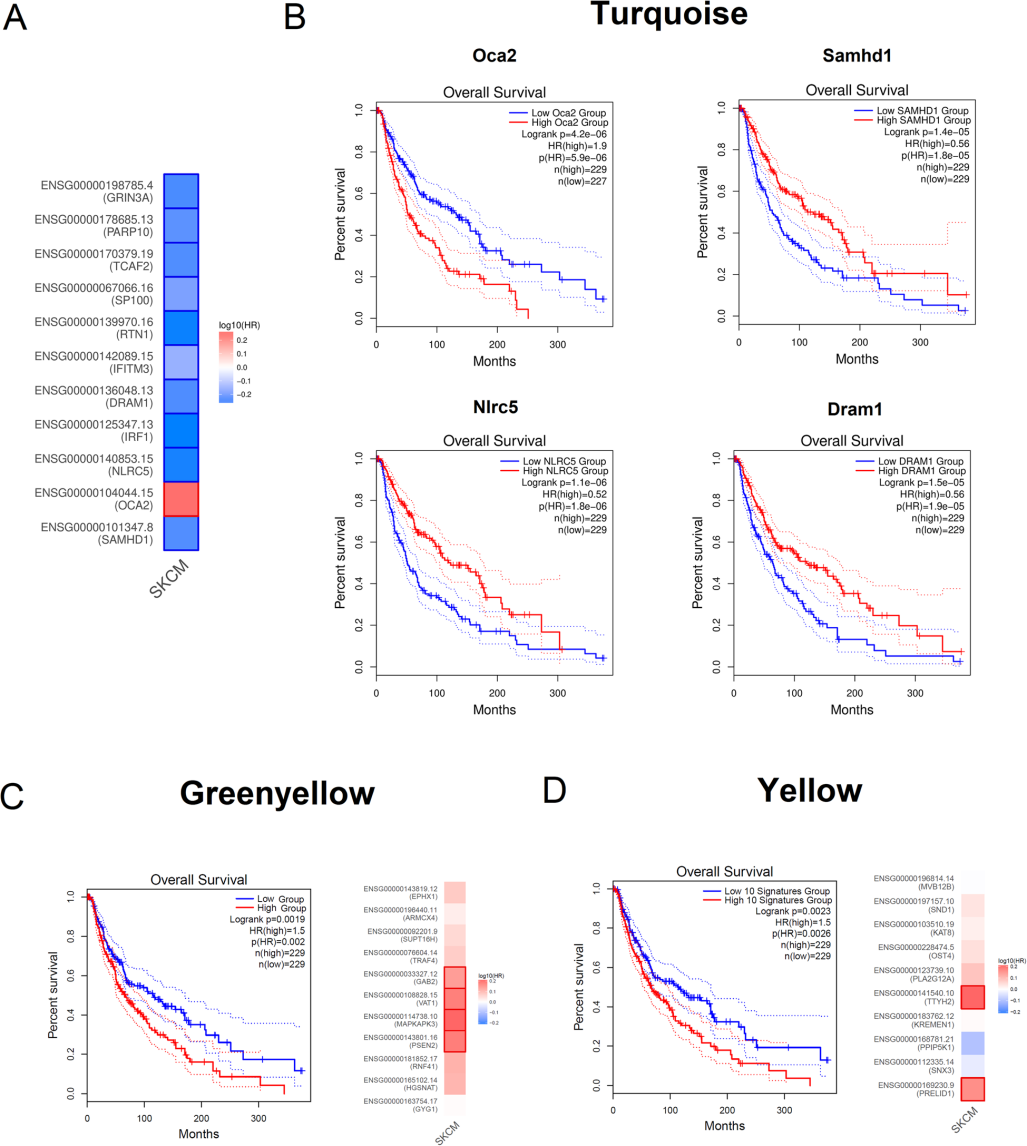
Relationship between the expression of genes in specific modules and overall survival in human melanoma patients from the TCGA melanoma cohort. The search for the prognostic value of genes in specific modules was performed using TCGA (The Cancer Genome Atlas) data for skin cutaneous melanoma (SKCM). Kaplan-Meier curves were built using overall survival in months as the clinical outcome and separating samples into two groups based on the median values for each gene (high and low expression groups). **a** Heatmaps showing the hazard ratios in logarithmic scale (log10) for different genes from the turquoise module. The red and blue blocks indicate higher and lower risks, respectively. The turquoise module had a significant enrichment (*p* = 0.009) of melanoma prognostic genes (500 top-ranked prognostic genes by *p* value) identified using the TCGA SKCM data. **b** Kaplan–Meier survival plots showing the prognostic value of the genes *Oca2*, *Samhd1*, *Nlrc5*, and *Dram1* from the turquoise module in skin cell melanoma samples from TCGA. *Oca2* showed a significant negative prognostic value in melanoma (i.e., higher expression was associated with worst prognosis), whereas *Samhd1*, *Nlrc5*, and *Dram1* had a positive prognostic impact. **c** Ten-gene hub signature from the green yellow module shows significant prognostic value (HR = 1.5, *p* value = 0.002) in human melanoma. The hub genes *Gab2*, *Vat1*, *Mapkapk3*, and *Psen2* also showed individual significant prognostic values. **d** Ten-gene hub signature from the yellow module shows significant prognostic value (HR = 1.5, *p* value = 0.002) in human melanoma. The hub genes *Ttyh2* and *Prelid1* also showed individual significant prognostic impact in melanoma. Genes or gene signatures with log-rank *p* values less than 0.05 were considered to have statistically significant prognostic value. The cox proportional hazard ratios and 95% confidence intervals were also included in the survival plots

The midnight blue, green yellow, yellow, blue, and light cyan modules showed a positive association with the metastatic cell line 4C11+ (Fig. 1b). The genes belonging to these modules exhibited enriched functions such as focal adhesion, insulin receptor signaling, MAPK activity, triglyceride biosynthesis, autophagosome organization, cytoskeleton organization, Ras signaling, ubiquitin-proteasome degradation, DNA damage response, mitochondrial translation, hippo pathway, and endocytosis (Table 2). In the subnetwork linked to metastasis (Fig. 2e), the hub genes from the green yellow and yellow modules were the most connected, such as *Supt16*, *Psen2*, *Gyg*, *Hgsnat*, *Vat1*, *Mapkapk3* (green yellow), and the genes *Prelid1*, *Snx3*, *Ost4*, *Mvb12b*, *Kat8*, *Pla2g2a*, and *Ttyh2* (yellow), followed by hubs from the blue, midnight blue, and light cyan modules. Among these hub genes, *Supt16* is a component of the FACT (facilitates chromatin transcription) complex, and *Kat8* codes for a lysine acetyltransferase that acetylates H4K16; both of which are altered in aggressive tumors [23, 24]. In the midnight blue module, the hub *Flt1* (fms-related tyrosine kinase 1) is a VEGF receptor that participates in the proliferation of melanoma cells [25], whereas *Cdh5* (VE-cadherin) is highly expressed in metastatic melanoma [26].

### Modules of co-expressed genes and their hubs are overrepresented by genes involved with poor prognosis in human melanoma

We have explored whether specific modules and hub genes identified in our murine model could be associated with overall patient survival using TCGA skin cell melanoma (SKCM) data. Three different approaches were taken. First, we verified if specific modules showed an overrepresentation of homolog genes selected from a list of 500 top-ranked genes (based on adjusted *p* values) whose expression levels displayed association with worse outcome in human melanoma cases. Hypergeometric distribution tests were applied to evaluate the enrichment of prognostic genes in the modules.

Only the module turquoise had a significant enrichment (*p* = 0.009) of genes whose homologs displayed prognostic value in melanoma, such as *Oca2*, *Samhd1*, *Nlrc5*, *Irf1*, *Ifitm3*, *Sp100*, *Dram1*, *Rtn1*, *Tcaf2*, *Parp10*, and *Grin3a* (Fig. 2a and b). Except for the melanocyte-specific protein *Oca2*, the expression of these genes was positively associated with overall survival in the TCGA SKCM data. This is in line with the negative correlation between the turquoise module and the metastatic trait in our cellular model.

*Samhd1*, for example, encodes a protein that regulates intracellular dNTP levels and DNA damage repair and shows tumor suppressive functions in many cancer types [27]. In parallel, the overexpression of the NOD-like receptor CARD domain-containing 5 (NLRC5) in melanoma cells enhances antitumor immunity by increasing the expression of MHC class I genes and presentation of tumor antigens [28]. Moreover, *Irf1*, *Ifitm3*, and Sp100 are involved in the interferon-mediated response that plays a role in antitumor immunity in melanoma [29, 30]. DRAM1 (DNA damage-regulated autophagy modulator 1) and RTN1 (reticulon 1) are autophagy-inducing factors [31]. Lastly, TCAF2 is a transient receptor potential channel-associated factor involved in melanoma cell death [32].

Secondly, we applied log-rank tests to check the prognostic value of 10-gene signatures comprising the hub genes in each module. For this analysis, a signature score was calculated for each patient, as the mean expression value of the homolog genes comprising the 10-gene signature. Next, the patients were ranked according to the signature score and stratified into high and low expression groups [33]. We observed that higher signature scores from hub genes of the green yellow (Fig. 2c, *p*= 0.002) and yellow (Fig. 2d, *p* = 0.002) modules showed a significant correlation with worse prognosis in human melanoma. These modules are highly correlated (Figure S2B) and participate in functions such as autophagy, endocytosis, cytoskeleton and mitochondria organization, Ras signal transduction, and Hippo signaling pathway (Table 2).

Individual relationships were also searched between the expression of homolog genes from hubs in each module and patient survival in the TCGA SKCM data. This analysis found 13 hub genes whose expression had prognostic value in human melanoma. These hubs belong to the modules green yellow (*Gab2*, *Mapkapk3*, *Psen2*, and *Vat1*), yellow (*Ttyh2*, *Prelid1*), tan (*Aco2*), light cyan (*Mks1* and *Commd3*), magenta (*Cars2*), black (*Psma3*), red (*Lrch3*), and midnight blue (*Bace2*). Among these hub genes, GAB2 is an adaptor protein commonly overexpressed in melanoma that upregulates RAS-ERK and PI3K-AKT pathways and promotes melanoma metastasis [34]. *Psen2* encodes the protein presenilin-2, a MYC target increased in melanoma cells [35], whereas *Mapkapk3* is a member of the p38 pathway that promotes autophagy [36]. TTYH2, in turn, is a volume-regulated anion channel that regulates transcription factors involved in EMT and is critical for the migration of osteosarcoma cells [37]. *Prelid1* is a prognostic gene in breast cancer that forms a complex in the mitochondrial intermembrane space to prevent apoptosis [38], and BACE2 (beta-secretase 2) plays a role in melanosome amyloid formation during melanocyte differentiation [39]. Finally, *Mks1* encodes the Meckel syndrome-associated protein that mediates ciliary trafficking; the loss of primary cilium was shown to drive melanoma metastasis via WNT/β-catenin signaling [40].

The enrichment of specific modules with prognostic genes was also explored using the Leeds Melanoma cohort data, which unlike the TCGA melanoma cohort includes only primary melanoma patients. Multivariate survival analyses (MA) showed that 553 genes were significantly associated with melanoma-specific survival after adjusting for age, sex, and tumor site (MA1), and this number was reduced to 276 genes when the AJCC (American Joint Committee on Cancer) stage and mitotic rate were further adjusted (MA2). Among the potential prognostic markers in the MA1 and MA2 survival models, 184 and 101 genes were also present in the co-expression modules, respectively.

**Fig. 3:**
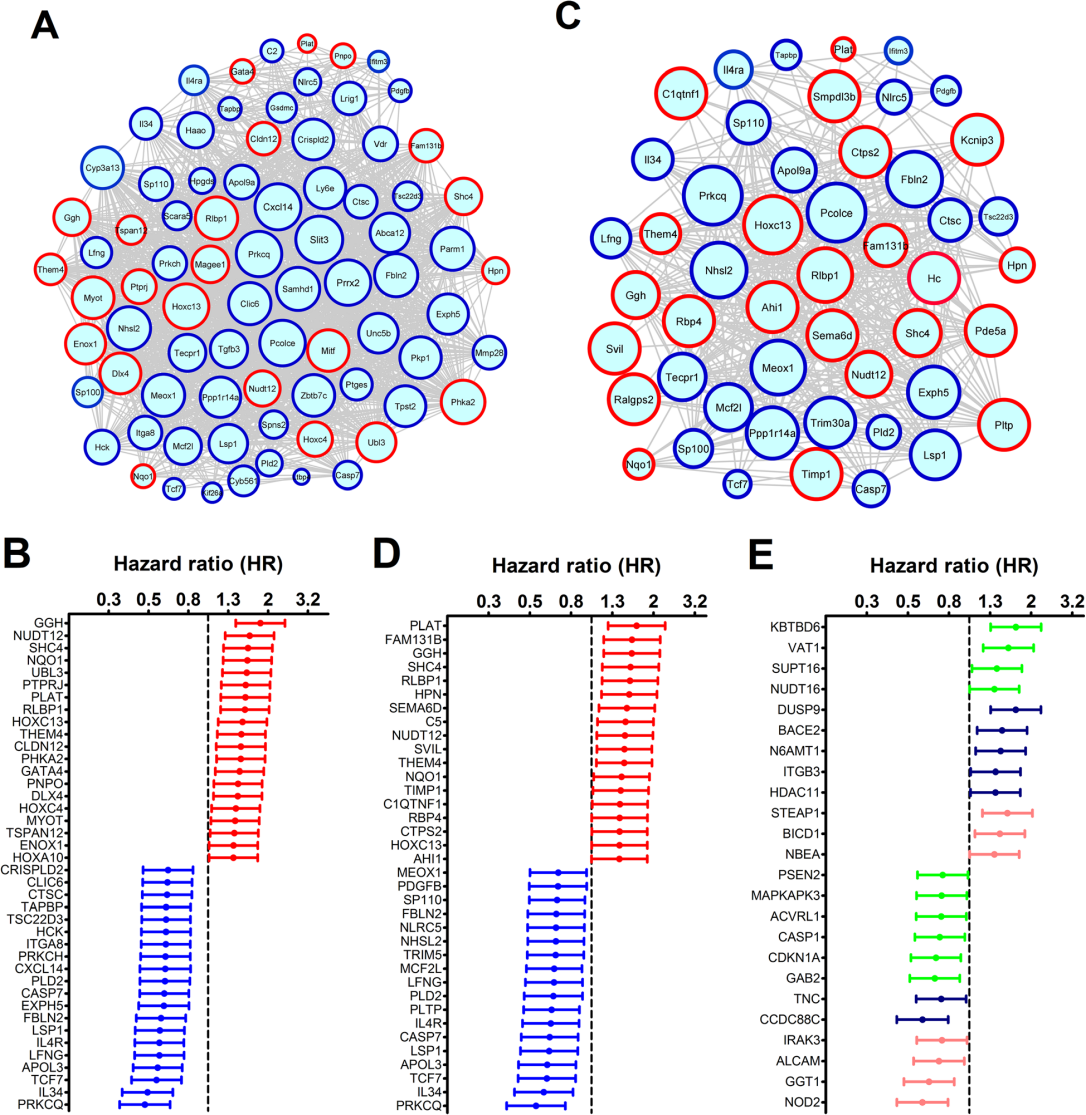
Co-expression interactions and hazard ratios for genes belonging to modules with prognostic value in the Leeds Melanoma cohort data. Multivariate analyses were performed by adjusting (i) age, sex, and tumor site (MA1 analysis) or (ii) as above plus AJCC stage and mitotic rate (MA2 analysis). Genes from the turquoise module were significantly overrepresented with genes with prognostic value in both MA1 (*n* = 80 genes, *p* = 2.4 × 10^−12^) and MA2 (*n* = 49 genes, *p* = 5.1 × 10^−10^) models. The green yellow module showed a significant overrepresentation of prognostic genes in the MA1 (*p* = 0.017, *n* = 10 genes), whereas the midnight blue and salmon modules showed a trend to be enriched with prognostic genes in the MA1 (*p* = 0.083, *n* = 7 genes; *p* = 0.060, *n* = 8 genes, respectively). **a** Co-expression network showing the relationships between the 80 genes from the turquoise module that were identified as prognostic genes in the MA1; the borders of the nodes representing genes with the highest (detrimental) and lowest (protective) hazard ratios were colored in red and blue, respectively. **b** Forest plot showing the hazard ratios (HR) and 95% confidence intervals for the genes from the turquoise module in the MA1; HR between 0 and 1 (protective) or larger than 1 (detrimental) are shown in blue and red colors, respectively. Circles represent the HR and the horizontal lines extend from the lower to the upper limit of the 95% confidence interval for the estimated HR. **c** Co-expression network showing the interactions between the 49 genes from the turquoise module that were identified as prognostic genes in the MA2. **d** Forest plot showing the hazard ratios (HR) and 95% confidence intervals for the genes from the turquoise module in the MA2; HR between 0 and 1 (protective) or larger than 1 (detrimental) are shown in blue and red colors, respectively. **e** Forest plot displaying the hazard ratios (HR) and 95% confidence intervals for the genes from the green yellow, midnight blue, and salmon modules in the MA1 model; the dots and lines were colored according to the module assignment of each gene for visualization purposes

The modules enriched with prognostic genes from the Leeds Melanoma Project data are in agreement with the analysis of the TCGA SKCM data. Genes from the turquoise module were significantly overrepresented among the prognostic genes both in the MA1 (*n* = 80 genes, *p* = 2.4 × 10^−12^) and MA2 (*n* = 49 genes, *p* = 5.1 × 10^−10^) models. Figure 3a and c show the co-expression interactions among the genes from the turquoise module that were present within the prognostic genes identified in MA1 and MA2, respectively. Figures 3b and d show the forest plots with the genes from the turquoise module that had significant (*p* value < 0.05) hazard ratios (HR) in MA1 and MA2, respectively. In both analyses, the genes with the highest HR (depicted in red) were *Plat*, *Ggh*, *Shc4*, *Them4*, *Nudt12*, *Rlbp1*, and *Hoxc13.* SHC4 is expressed during the transition to metastatic melanoma [41], NUDT12 (Nudix-type motif 12) is a member of the NUDIX hydrolase family involved in cancer cell metabolism and survival [42], and GGH (γ-glutamyl hydrolase) regulates intracellular folate levels that are necessary for DNA replication. Conversely, the genes with the lowest HR (shown in blue) were *Prkcq*, *Il34*, *Tcf7*, *Apol9a*, *Lfng*, *Il4r*, *Lsp1*, Casp7, *Fbl2*, and *Pld2*. Among them, there were some genes involved in immune-related functions (*Il34*, *Tcf7*, *Il4r*, and *Lsp1*). Other genes are related to apoptosis (*Casp7*) and mitotic arrest (*Fbl2*) [43]. In addition, *Lfng* (Lunatic fringe) encodes a glycosyltransferase that inhibits metastasis in melanoma cells [44].

**Fig. 4:**
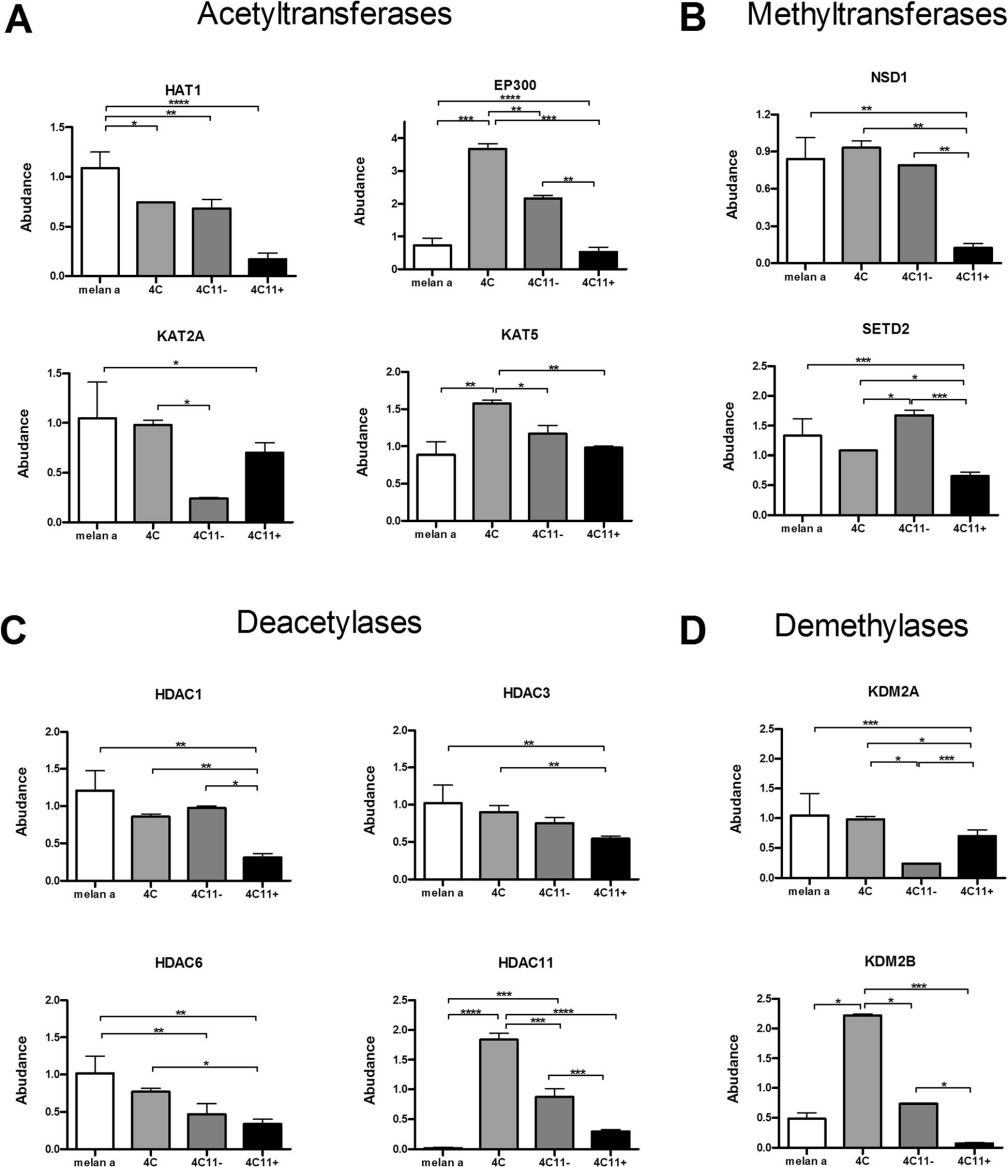
Comparative gene expression analysis of histone writers and erasers across the cell lines from the melanoma progression model. Gene expression levels were assessed by quantitative polymerase reaction (qPCR) analysis of the cell lines melan-a, 4C, 4C11−, and 4C11+. **a** Gene expression analysis for the lysine acetyltransferases *Hat1*, *Ep300*, *Kat2a*, and *Kat5*. *Hat1* showed the highest expression in melan-a cells and then exhibited progressive lower expression in the 4C, 4C11−, and 4C11+ cell lines. *Ep300* showed statistically significant upregulation in the mesenchymal-like cell lines 4C and 4C11−. *Kat5* was significantly upregulated in 4C cells in comparison to melan-a cells but returned to melan-a levels in 4C11− and 4C11+ cells. **b** Gene expression analysis for the lysine methyltransferases NSD1 and SET2. The gene expression of *Nsd1* was significantly downregulated in 4C11+ cells in comparison to the other cell lines. *Setd2* expression was the highest in melan-a and 4C11− cells, showing intermediate values in 4C cells and the lowest expression in 4C11+ cells. **c** Gene expression analysis for the histone deacetylases HDAC1, HDAC3, HDAC6, and HDAC11. The expression levels of *Hdac1*, *Hdac3*, and *Hdac6* were progressively reduced in the cell lines, with 4C11+ cells showing the lowest expression levels. In contrast, the expression levels of *Hdac11* were higher in both mesenchymal-like cell lines 4C and 4C11−. **d** Gene expression analysis for the histone demethylases *Kdm2a* and *Kdm2b*. The expression of *Kdm2a* was significantly reduced in 4C11− cells. *Kdm2b* expression was significantly increased in 4C cells compared to melan-a cells and decreased in 4C11− and 4C11+ cells. Statistical analyses were made by comparing the expression levels of each gene across the cell lines using one-way ANOVA followed by the Tukey’s multiple comparison test. *p < 0.05, **p < 0.01, and ***p < 0.001. Genes were considered to be differentially expressed when the multiplicity adjusted *p* values in the pairwise comparisons were lower than 0.05

The green yellow module showed a significant overrepresentation of prognostic genes only in the MA1 (*p* = 0.017, *n* = 10 genes), with the genes *Gab2*, *Vat1*, *Mapkapk3*, *Psen2*, *Supt16*, *Casp1*, *Kbtbd6*, *Cdkn1a*, *Nudt16*, and *Acvrl1* (Fig. 3e); the first five genes from this list are hubs in this module. The midnight blue and salmon modules also had a trend toward significant enrichment of prognostic genes in the MA1 model (*p* = 0.083, *n* = 7 genes; *p* = 0.060, *n* = 8 genes, respectively). The genes *Ccdc88c*, *Bace2*, *N6amt1*, *Dusp9*, *Itgb3*, *Tnc*, and *Hdac11* from the midnight blue module were prognostic markers in MA1 (Fig. 3e). *Ccdc88c* is a hub gene whose expression in circulating tumor cells is associated with melanoma survival [45]. In parallel, the gene expression of *Hdac11* was significantly higher in mesenchymal (4C and 4C11−) versus epithelial (melan-a and 4C11+) cells (Fig. 4c). The genes *Pcdhb18*, *Bicd1*, *Nod2*, *Nbea*, *Steap1*, *Irak3*, *Alcam*, and *Ggt1* in the salmon module had prognostic value in MA1. *Bicd1* (BICD cargo adaptor 1) is a hub gene in this module, and its expression is strongly correlated with EMT and worse prognosis in glioblastoma [46].

### The transcriptional modules are significantly enriched with genes whose expression is regulated by histone modifications

The identification of hub genes associated with the epigenetic machinery prompted us to map changes in histone modification enzymes in the gene co-expression modules. Hypergeometric tests were applied in each module to assess if any particular module would be enriched with genes encoding proteins involved in histone modification (*n* = 438 genes, GO 0016570). Among the genes present in this GO term, 126 were identified in the co-expression modules. Only the brown module was significantly overrepresented with genes associated with histone modification (24 genes, *p* = 0.0024). For instance, the genes *Kdm4a* and *Kdm7a* (histone demethylases), *Hdac6* (histone deacetylase), and *Setd2* (histone methyltransferase) were present in the brown module. This module is the most dissimilar among the modules in the gene co-expression network (Figure S2B), suggesting that the expression of histone modification-related genes could be regulated independently from the other genes in the network. Moreover, the positive correlation of the brown module with the 4C cell line (Fig. 1a), although not statistically significant, may indicate the role of the genes in this module in melanoma initiation.

**Fig. 5:**
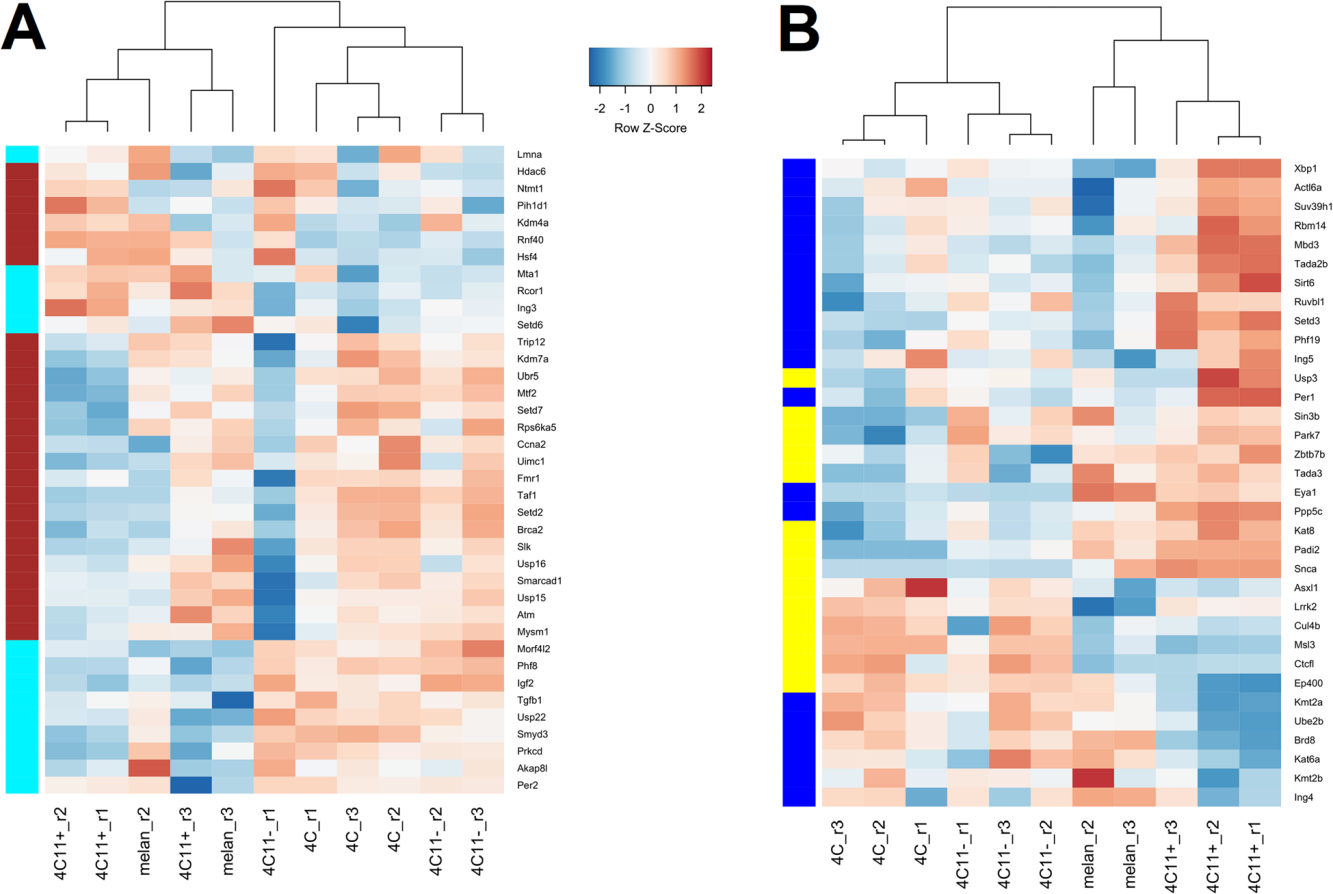
Hierarchical clustering of the samples from the cellular model of melanoma progression based on the expression of histone-modifying genes in the brown, turquoise, blue, and yellow modules. Genes involved in histone modification (GO term 00165700) were searched among those present in each module. One hundred twenty-six (126) genes associated with histone modification were found in the co-expression modules. The brown module was significantly enriched with these genes (*n* = 24 genes) and the turquoise, blue, and yellow modules also displayed a high number of histone modification genes, with fourteen, twenty, and fourteen genes, respectively. The Pearson correlation and the complete method were used as the distance metric and linkage method, respectively. Data is represented as *z* scores, calculated by subtracting the mean and dividing by the standard deviation of the row. The legend shows the color mapping of the row-wise *z* scores with blue and red colors. The epithelial (melan-a and 4C11+) and mesenchymal (4C and 4C11−) cell lines showed a trend to cluster together based on the expression of genes encoding histone-modifying proteins. **a** Hierarchical clustering of the samples and histone modification genes from the brown and turquoise modules, showing the most frequent downregulation of these genes in the metastasis-prone 4C11+ cell line. **b** Hierarchical clustering of the samples and histone modification genes from the blue and yellow modules, showing that these genes were most often upregulated in the samples from the 4C11+ cell line. The module assignment of each gene is shown on the left of the figures by their representative colors.

The turquoise, blue, and yellow modules also had a higher number of genes encoding histone-modifying enzymes, with fourteen, twenty, and fourteen genes respectively. Moreover, the blue and yellow modules showed a trend toward significant enrichment of histone modification genes (*p* = 0.096 and *p* = 0.113, respectively). The hierarchical clustering of the samples based on the expression of the histone modification genes from the brown and turquoise (Fig. 5a), or from the blue and yellow (Fig. 5b) modules, demonstrate that samples tend to cluster based on their epithelial (melan-a and 4C11+) or mesenchymal phenotypes (4C and 4C11−). The expression of these genes in the brown and turquoise modules is more frequently downregulated in the metastasis-prone 4C11+ cells, such as the genes *Taf1*, *Setd2*, *Setd7*, *Brca2*, *Mtf2*, *Per2*, and *Phf8* (Fig. 5a). MTF2 (metal regulatory transcription factor 2), for example, was shown to induce EMT by transcriptionally activating Snail in hepatocellular carcinoma, whereas PHF8 (PHD finger protein 8) is an enzyme that demethylates the H3K9, H3K27, and H4K20 lysine residues [47]. In contrast, histone modification genes from the blue and yellow modules (Fig. 5b) were more often upregulated in this cell line, such as *Per1*, *Sirt6*, *Padi2*, *Mbd3*, *Suv39h1*, and *Kat8*. MBD3 is a component of the NuRD complex that regulates epithelial–mesenchymal plasticity and tumor metastasis [48]. Finally, SUV39H1 is an enzyme that trimethylates H3K9 [5].

We also computationally searched for histone modifications that could regulate the transcriptional patterns in each module, by detecting overrepresented gene signatures previously associated with histone marks. Interestingly, genes related to histone modifications at H3K27 (H3K27ac and H3K27me3), H3K79 (H3K79me2 and H3K79me3), H3K4 (H3K4me3), H3K9ac, H3K36me3, and H4K20me1 were enriched in some modules (Table 2). The brown and blue modules showed more enriched histone marks: the brown module was involved with the H3K79me2, H3K27ac, HeK4me3, H3ac, H3K9ac, and H3K36me3 marks, and the blue module was enriched with genes regulated by the H3K79me3, H3K79me2, H3K27ac, H3K4me3, H3K36me3, and H4K20me1 marks. For instance, the hub *Kmt2a* (lysine methyltransferase 2A) from the blue module (Fig. 1c, Table 1) is responsible for H3K4 methylation and associated with melanoma growth [49]. Other gene modules directly or inversely correlated with metastasis were enriched with the histone marks H3K4me3 (magenta and yellow), H3K36me3 (magenta, blue, yellow) and H3K79me2 (black, green yellow, light cyan), and H3K27ac (blue, black, magenta).

### The abundance levels of clusters of combinatorial histone marks are related to melanoma progression, EMT, and metastasis

To further explore the connection between chromatin remodeling and melanoma progression, the status of several histone PTMs was experimentally determined by a high-resolution workflow that employs chemical derivatization and mass spectrometry analysis of histone residues. The abundance ratios of 245 histone PTM peptides (containing single or multiple/combined PTMs) were determined in pairwise comparisons across the cell lines (Tables S1 and S2). The Pearson correlation of the biological replicates’ abundance ratios indicates a good correlation among them (Figure S3). In addition, the levels of histone PTMs were overall less correlated in the 4C11+ replicates compared to the other cell lines.

**Fig. 6:**
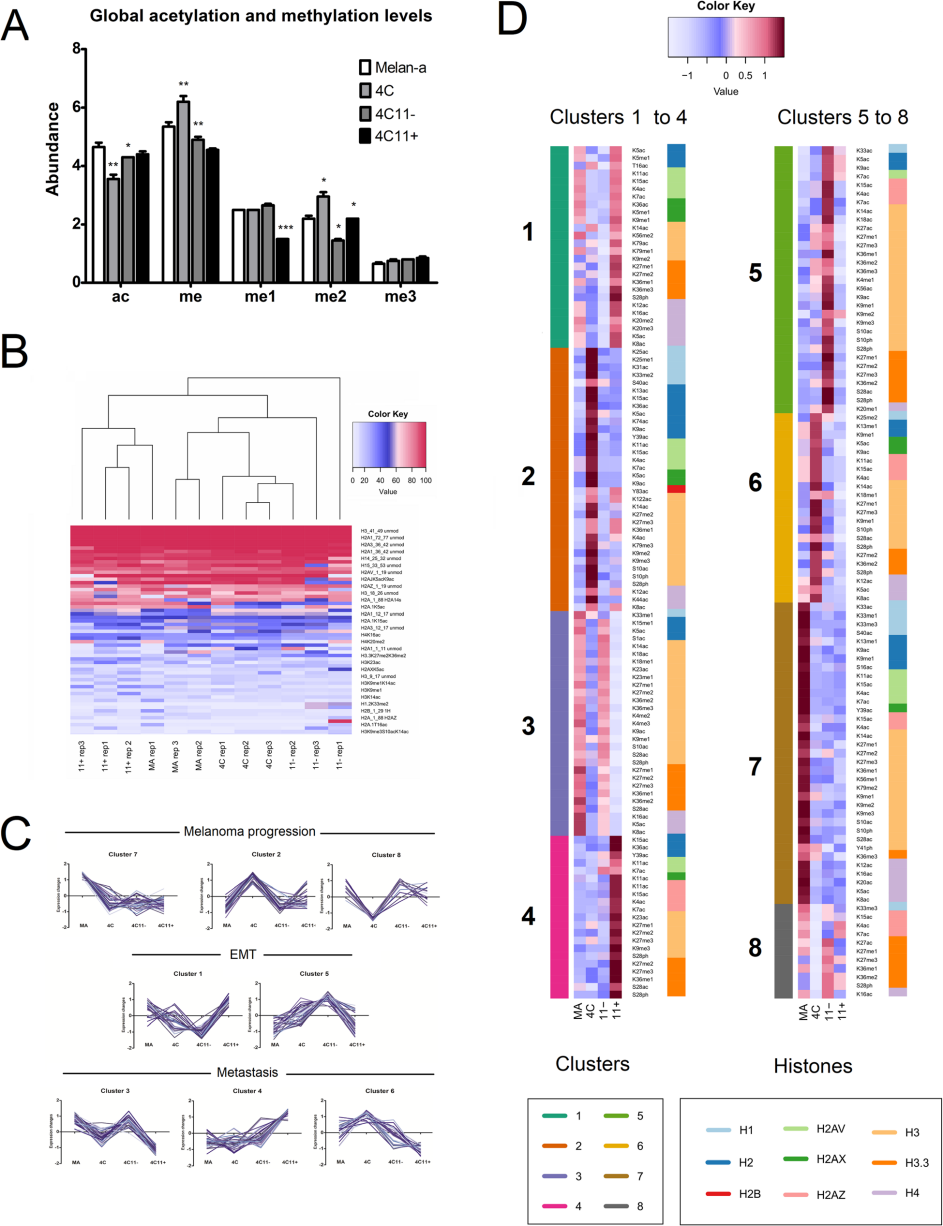
Analysis of global and combinatorial histone modifications (PTMs) during melanoma progression. The acetylation and methylation levels of histone marks were assessed in the cell lines from the melanoma progression model. **a** Global acetylation and methylation (me1 + me2 + me3) levels were obtained by the sum of the abundance ratios of all peptides containing these modifications. The 4C cell line had a decrease in global acetylation levels and an increase in the dimethylation and global methylation histone marks. **b** Hierarchical clustering of the samples was based on the relative abundance levels of 245 histone combinatorial PTMs, using the Pearson correlation and the complete linkage method. The 65 histone marks with the highest relative abundance levels are shown. Cell lines with epithelial (4C11+ and MA) and mesenchymal (4C and 4C11−) morphology cluster together. **c** c-fuzzy means clusters of histone combinatorial PTMs were grouped according to their potential role in melanoma progression, EMT, and metastasis. The clusters 2, 7, and 8 were associated with melanoma progression due to the alteration of their abundance distribution in the 4C, 4C11−, and 4C11+ cell lines. Clusters 1 and 5 were related to epithelial-mesenchymal transition (EMT) due to their relative abundance patterns in epithelial versus mesenchymal cells. Clusters 3, 4, and 6 were associated with metastasis due to the increased or decreased regulation of the histone peptide marks in these clusters in the metastasis-prone 4C11+ cell line. **d** Heatmaps showing the changes in the relative abundance levels of the combinatorial PTMs grouped in each cluster. Since modifications of the same amino acid residues occur in multiple peptides and clusters, only the average *z* scores are reported for each histone residue-modification pair in each cluster. The color mapping of the row-wise *z* scores was made with blue and red colors to represent the down- or upregulation of the corresponding PTMs in each cell line. The row colors on the left side of each heatmap illustrate the clusters in which the combinatorial PTMs are included, whereas the row colors on the right side depict the histones from which the modified amino acid residues belong to

First, the global acetylation and methylation relative abundance levels of all histone peptides were compared across the cell lines. Interestingly, global acetylation levels drop in the 4C cell line whereas methylation levels are increased (Fig. 6a), suggesting that major histone modification events may occur in the initial stages of melanoma progression. In general, most histone PTMs were present at low abundance in all cell lines, except by some PTMs at histones H4 (K16ac and K20me1), H3.3 (K27me), H3.1 (K4me1, K14ac, K23ac, K27me), and H2A.1 (K5ac, K15ac) (Fig. 6b) (Table S2). The hierarchical clustering based on the combinatorial histone PTMs revealed that samples from the cell lines with epithelial (4C11+ and MA) and mesenchymal (4C and 4C11−) morphology clustered together (Fig. 6b).

To gain further insights into the pattern of global histone PTM changes during melanoma progression, we grouped histone combinatorial marks using a c-fuzzy mean clustering algorithm. This clustering method allows one histone PTM to belong to multiple clusters, which may be relevant to find common and exclusive histone marks involved in melanoma progression, EMT, or metastasis. Eight clusters were found, which were further classified as being associated to tumor progression (clusters 2, 7, and 8), EMT (clusters 1 and 5), and metastasis (clusters 3, 4, and 6), based on their relative abundance patterns across the cell lines (Fig. 6c and Table S3). Heatmaps were built to visualize the changes in the relative abundance levels of the combinatorial PTMs grouped in each cluster, and to show which histones and modified residues belong to each cluster (Fig. 6d). Since modifications of the same amino acid residues occur in multiple peptides, we reported the average *z* scores for each histone residue-modification pair in each cluster in Fig. 6d.

The clusters 2, 7, and 8 contain histone PTMs whose levels start to change in the 4C cell line (Fig. 6d), pointing to specific histone alterations potentially involved in melanoma initiation, such as acetylation changes in H3K4, H4K12, and H4K16, as well as the methylation of H3K9 and H3K27 residues. In particular, the cluster 7 comprises histone PTMs whose levels decrease upon melan-a cell transformation to 4C cells (Table S3). It contains several acetylation marks at the H4 N-terminus (lysine residues 5, 8, 12, 16, and 20), further suggesting that the deacetylation of H4 lysine residues may be an epigenetic mechanism involved in melanoma progression.

The clusters 1 and 5 encompassed histone PTMs whose relative abundance levels displayed a differential pattern between the epithelial (melan-a and 4C11+) and mesenchymal (4C and 4C11−) cell lines (Fig. 6c and d). Histone PTMs in the H3K9, H3.3K27, H3K56, H3K79, H4K20, and histone H2A variants (H2AV, H2AX, and H2AZ) lysine residues were observed in these clusters.

Finally, multiple combinatorial methylation and acetylation marks at H3K9, H3K14, H3K27, and H3K36 were found in clusters associated with metastasis in our model (clusters 3, 4, and 6). Reduced acetylation of H3K14 was seen in several histone peptides from the 4C11+ cell line (Table S3), suggesting a potential role of H3K14ac in metastasis. In contrast, the acetylation levels of amino acid residues from the histones H2A1, H2AZ, and H2B were upregulated in the 4C11+ cell line (cluster 4, Fig. 6d).

### The levels of single histone H3 and H4 marks and genes encoding histone-modifying enzymes are related to melanoma progression, EMT, and metastasis

**Fig. 7:**
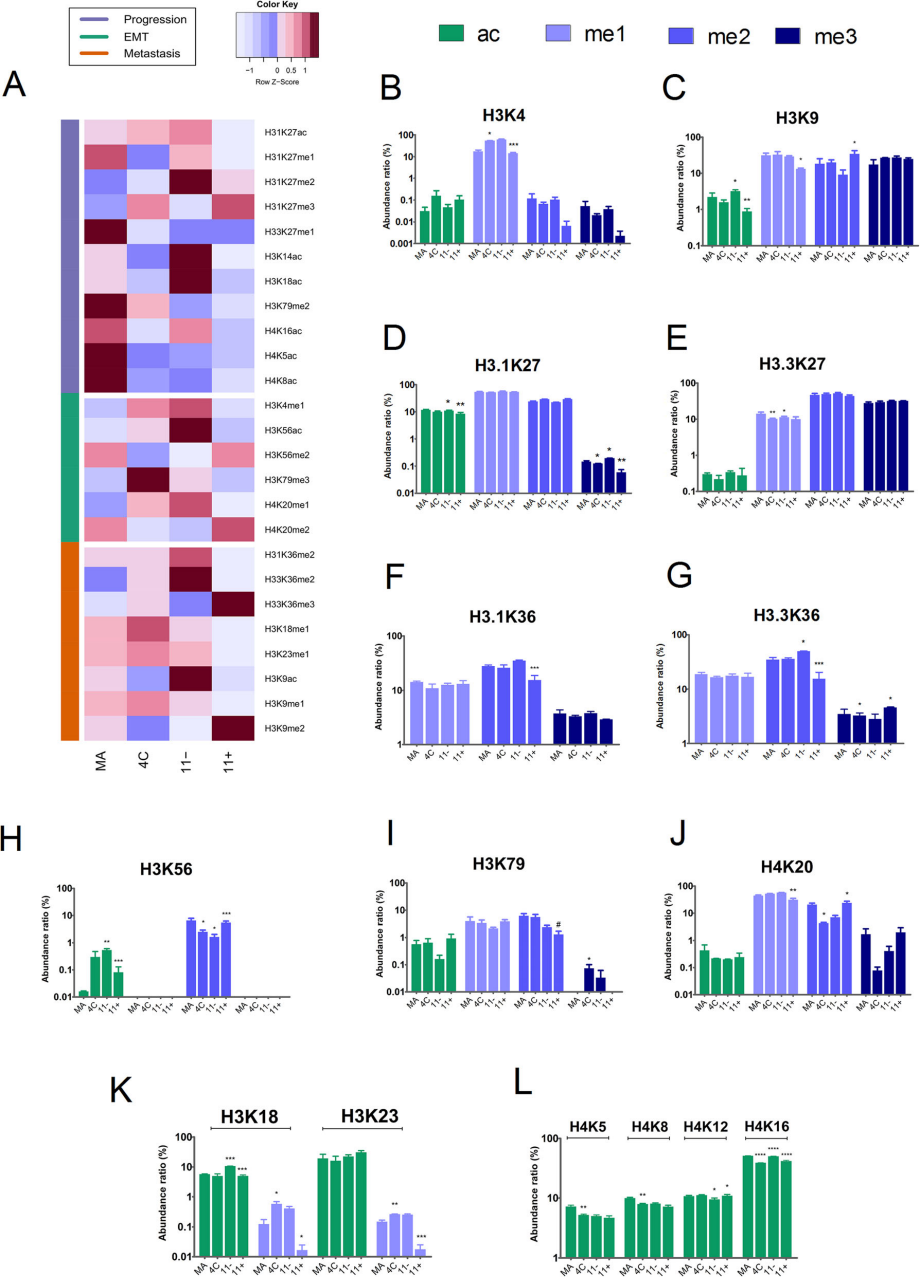
Single post-translational modifications (PTMs) in histones H3 and H4 are associated with melanoma progression, EMT, and metastasis. **a** Heatmap showing the changes in the average abundance levels of specific single histone marks across the cell lines, and their potential association with melanoma progression, EMT, and metastasis. The associations were made based on the relative abundance patterns of the single histone marks across the cell lines. The levels of histone PTMs were statistically compared using one-way ANOVA followed by Tukey’s multiple comparison tests. Differences were considered statistically significant when the multiplicity adjusted *p* values were lower than 0.05. Only single histone PTMs that were differentially expressed in at least one pairwise statistical comparison are shown. Significant pairwise comparisons between melan-a versus 4C, 4C versus 4C11−, and 4C11− and 4C11+ cells are shown as *p < 0.05, **p < 0.01, and ***p < 0.001, whereas the significant difference between melan-a versus 4C11+ for H3K79me2 is shown as ^#^*p* < 0.05. **b** The levels of H3K4me1 are decreased in mesenchymal (4C and 4C11−) compared to epithelial cell lines (melan-a and 4C11+). **c** The dimethylation levels at H3K9 were increased in the metastasis-promoting 4C11+ cells compared to the non-metastatic 4C11− cells, whereas the acetylation levels of H3K9 were decreased in 4C11+ cells. **d**, **e** For H3K27, the H3.1K27me3 and H3.3K27me1 marks were differentially altered in the cell lines. **f**, **g** The dimethylation levels of H3K36 (H3.1K36 and H3.3K36) were reduced in 4C11+ cells, but the H3.3K36me3 mark was increased in this cell line. **h** The H3K56me2 mark was significantly increased in the mesenchymal cell lines 4C and 4C11−. **i** The H3K79me2 was progressively reduced across the cell lines, with significant changes between the melan-a and 4C11+ cell lines. **j** The methylation levels of H4K20 were associated with EMT, with the H4K20me2 levels being significantly decreased in the mesenchymal cell lines. **k** The acetylation and monomethylation levels of H3K18 and H3K23 were statistically different in the cell lines and related to melanoma progression. **l** The acetylation levels of H4K5, H4K8, H4K12, and H4K16 were statistically different across the cell lines. Lower levels of H4K5 and H4K8 acetylation are seen in 4C, 4C11−, and 4C11+ in comparison to melan-a cells. In parallel, the H4K12ac and H4K16ac changed across the multiple pairwise comparisons

We also examined in details the abundance levels of a subset of 45 single PTMs from histones H3 and H4. Statistical comparisons using one-way ANOVA followed by Tukey’s multiple comparison tests were performed to detect single histone marks with differential levels between the cell lines (adjusted *p* values are reported in Table S4). Remarkably, the number of alterations in histone PTMs seems to increase in the path toward melanoma metastasis in our model, since the comparisons against the 4C11+ cells led to more statistically significant differences (Table S5). Figure 7a displays a heatmap containing the single histone marks that had differential abundance levels across the cell lines, and their potential association with melanoma progression, EMT, and metastasis.

The levels of H3K4me1 (Fig. 7b), H4K20me1 (Fig. 7j), H3K56ac (Fig. 7h), and H3K79me3 (Fig. 7i) modifications were significantly increased, whereas H3.3K36me3 (Fig. 7g), H3K56me2 (Fig. 7h), and H4K20me2 (Fig. 7j) marks were decreased in mesenchymal (4C and 4C11−) versus epithelial cell lines (melan-a and 4C11+). The increased H3K56 acetylation in the mesenchymal cell lines (4C and 4C11−) is in line with the significant upregulation of *Ep300* in the same cells (Fig. 4a). Moreover, a higher expression of the H3K36 demethylase *Kdm2b* was also observed in the mesenchymal cell lines (Fig. 4d), followed by a downregulation in 4C11+ cells.

Patterns of histone modifications were also associated with melanoma progression and metastasis. Melan-a cells showed high levels of H4K5ac and H4K8ac marks (Fig. 7l) and increased expression of *Hat1* (Fig. 4a), which catalyzes H4K5 acetylation. H3K79me2 levels were also progressively reduced in the model (Fig. 7i). In addition, the levels of the H3K9me2 (Fig. 7c) and H3.1K27me3 (Fig. 7d) repressive marks were significantly altered in the metastasis-prone 4C11+ cells compared to the non-metastatic 4C11− cells. This decreased pattern was also seen in the 4C11+ cell line for the monomethylation of H3K18 and H3K23 and the acetylation status of H3K18 (Fig. 7k).

The H3K36 dimethylation levels (Fig. 7f–g) were decreased in 4C11+ cells. The increase in the H3K36me2 levels in the 4C11− cells is in agreement with the reduced gene expression of the H3K36 demethylase *Kdm2a* (Fig. 4d) in this cell line. Conversely, the decrease in the H3K36me2 mark in 4C11+ cells is consistent with the reduced gene expression of *Nsd1* (Fig. 4b) that dimethylates H3K36 [5]. Finally, the increased trimethylation of H3.3K36 in 4C11+ cells (Fig. 7g), which is an epigenetic marker associated with transcription activation, highlights the role of H3K36me3 deregulation in the path toward metastasis.

## Discussion

In the present study, we have characterized gene co-expression and histone modification signatures associated with melanoma progression and metastasis in a four-stage cellular model of melanoma. The current surveillance strategy of melanoma patients is based on the AJCC classification, but still many patients develop the metastatic disease despite diagnosed in early stages, suggesting that some tumors could have a higher propensity to metastasize [50]. Therefore, the unraveling of molecular mechanisms that drive melanoma aggressiveness is crucial to improve clinical outcomes.

The co-expression network analysis identified transcriptional modules related to melanoma progression, EMT, and metastasis. The genes in these modules participate in functions such as focal adhesion, cell migration, extracellular matrix organization, endocytosis, cell cycle, DNA repair, protein ubiquitination, and autophagy. Among these functions, ubiquitination is an emerging regulator of tumor promoters and suppressors in the pathogenesis of melanoma [51], whereas autophagy has been recognized as tumor-suppressive in the early stages of melanoma but tumor promoting in established tumors [52].

Co-expression modules that were highly correlated to EMT and metastasis (turquoise, green yellow, and yellow) in the model were also enriched with prognostic genes identified from two independent melanoma cohorts (TCGA and Leeds). This finding highlights the relevance of exploring such transcriptional modules and their hub genes as potential diagnostic biomarkers of melanoma aggressiveness. Previous studies have also demonstrated the potential of identifying genes involved in melanoma metastasis by means of analyzing co-expression modules related to the metastatic trait [53, 54].

Several activation and repression histone marks were predicted to be enriched in the co-expression modules, and their differential levels were experimentally confirmed by mass spectrometry. The epigenomic profiling of more than two hundred histone modifications allowed the identification of clusters of combinational marks and single PTMs that were associated with tumor progression, EMT, or metastasis.

The methylation of H3K4, H3K56, and H4K20 and the acetylation of H2A.Z residues were related to EMT. For example, enrichment of H3K4me1 is gradually increased over the course of Snail-induced EMT [55], and the levels of H3K4me3 are altered close to genes from melanoma-driving pathways [56]. The increased H3K56ac in the mesenchymal cell lines (4C and 4C11−) is in line with the significant upregulation of *Ep300* in the same cells. The Ep300-mediated acetylation of H3K56 participates in the DNA damage response [57]. In addition, the decrease in H4K20 trimethylation promotes EMT and cell migration in breast cancer [58]. The H4K20 methylation state is relevant not only for chromatin compaction but also for DNA repair; cells deficient to KMT5B/C, responsible for the di- and tri-methylation of H4K20 respectively, have increased sensitivity to DNA damage [59]. Finally, the histone variant H2A.Z is a master regulator of EMT that coordinates the expression of both epithelial and mesenchymal genes [60]. In particular, the H2A.Z isoform H2A.Z.2 is highly expressed in melanoma and increases cell proliferation by recruiting BRD2 and E2F1 to E2F target genes [61].

Other histone modifications were associated with melanoma progression and metastasis. Reduced acetylation of H4K5 and H4K8 were seen in the pre-malignant and tumorigenic cell lines. In addition, the metastatic 4C11+ cell line exhibited higher H3K9me2 and H3K36me3 methylation but lower H3K18ac, H3K18me1, H3K23me1, H3K79me2, and H3K36me2 marks. The decrease in H4 acetylation and H3K79me2 levels points to the association of these histone marks with melanoma progression. The loss of acetylation marks at H4 may play a role in melanoma initiation [56], due to the silencing of tumor suppressor genes [62] or impairment of DNA repair [63]. Moreover, loss of H3K79me2 levels is involved in genomic defects during mitosis [64]. In fact, the gene-encoding DOT1L, a histone methyltransferase required for H3K79 methylation, is frequently mutated in human melanoma. DOT1L plays an important protective role in ultraviolet radiation-induced melanomagenesis [65]. H3K9me2 levels were also found to be increased in melanoma compared to normal skin tissue [66] whereas H3K27me3 levels were lower in metastatic versus primary melanoma cases [67]. In parallel, low H3K18ac levels in cancer were already correlated with poor patient outcomes [68] and the decrease in H3K23me1 was also verified in breast cancer when compared to normal tissues [69].

The reduced H3K36me2 but increased H3K36me3 levels in the metastatic 4C11+ cell line are in agreement with the correlation of these marks in colorectal cancer with poor prognosis and lymph node metastasis, respectively [70]. The regulation of H3K36me2 was recently involved with metastatic progression and mesenchymal state during EMT [71]. The increased expression of the H3K36 demethylase Kdm2b in the mesenchymal cell lines, followed by a downregulation in 4C11+ cells, points to the involvement of this enzyme in the decreased H3.3K36me3 levels during EMT. The H3K36 trimethylation also plays a role in homologous recombination repair, and consequently reduced H3K36me3 is associated with DNA damage [72].

## Conclusion

We have identified clinically meaningful modules of co-expressed genes that were associated with EMT and metastasis in a cellular model of melanoma. These modules were significantly enriched in genes associated to patient survival in two independent melanoma datasets. The genes in these modules also participate in biological processes such as focal adhesion, autophagy, cytoskeleton organization, ubiquitin-proteasome degradation, DNA damage response, and endocytosis. These co-expression patterns could serve as a basis for future research toward the discovery of novel biomarkers and functions that impact melanoma survival.

To our knowledge, this study also evaluates for the first time the genome-wide dynamics of histone PTMs along melanoma progression using an unbiased approach. The analysis of the combinatorial PTM changes in the cell lines revealed eight clusters of combinatorial marks that were associated with melanoma progression, EMT, or metastasis. Moreover, the examination of single marks from histones H3 and H4 disclosed that the reduced acetylation of H4K5 and H4K8 was linked to melanoma initiation in our model. On the other hand, the methylation levels of H3K4, H3K56, and H4K20 were associated with EMT. Lastly, the metastasis-prone 4C11+ cells had the highest number of differentially abundant histone marks: it exhibited higher H3K9me2 and H3K36me3 but lower H3K18me1, H3K23me1, H3K79me2 and H3K36me2 marks. Collectively, these findings contribute to the characterization of the gene co-expression patterns, biological functions, and histone PTMs that underlie melanoma progression and metastasis. Such molecular events may shape chromatin structure, silence tumor suppressor genes, impair DNA repair, induce EMT, and increase proliferation of melanoma cells.

## Methods

### Cellular model of melanoma initiation and progression

The murine cell model of melanoma initiation and progression was previously established by our group [9, 10]. The model consists of cell lines with increasing malignancy potential (4C, 4C11−, and 4C11+) that were obtained through sequential detachment/re-adhesion cycles from the non-tumorigenic melanocyte lineage melan-a, which is a spontaneously immortalized nonmalignant cell line syngeneic to C57Bl/6 mice [73]. The melan-a and 4C11+ cell lines exhibit an epithelial morphology while the intermediate 4C and 4C11− cells have a mesenchymal phenotype. The tumor 4C11− and 4C11+ cell lines show distinct metastasis-promoting potential; only 4C11+ cells generate lung metastasis in mice [11]. Cells were grown in RPMI medium pH 6.9 (Gibco, Carlsbad, CA) supplemented with 5% FBS and 1% penicillin and streptomycin at 37 °C under 5% CO_2_. The PMA (Phorbol Myristate Acetate, 200 nM) solution was added to the medium only for melanocytes melan-a.

### Baseline transcriptome sequencing data from the cell lines

The RNA sequencing data was analyzed as described elsewhere (Pessoa et al., submitted). Briefly, total RNA was isolated from triplicates of each of the four cell lines, and DNA libraries were prepared using 1 μg of total RNA and the Illumina TruSeq™ Stranded Total RNA Library Prep Kit with Ribo-Zero Gold (cat. no. RS-122-2001, Illumina Inc.). After quality control and adaptor trimming, the sequencing data was mapped to the annotated mouse genome (Ensembl 90 version) using the STAR aligner [74], and reads were mapped to genes using the Rsubread R package [75]. Only genes detected by at least one read count per million in at least four libraries were considered. Count values were normalized using the *voom* function in the *limma* R package [76]. The RNAseq data is available in GEO under the accession number GSE149884.

### Weighted gene co-expression network analysis

The WGCNA algorithm was implemented using the WGCNA package in R [77]. The WGCNA algorithm calculates pairwise absolute correlation coefficients from the expression data and raises the adjacency matrix values to a soft threshold, β. The β threshold is selected to maximize the scale-free topology of the resulting network. The soft threshold of 10 was chosen to reduce the mean connectivity of each node while maximizing the scale-free topology fit (Figure S1).

The WGCNA was performed to find modules of co-expressed genes, identify hub genes, and associate specific modules with cell lines or phenotypes (i.e., tumor behavior, epithelial or mesenchymal states, metastasis potential). Only the 5000 most variable genes across the cell lines, measured by their variance, were analyzed to reduce background noise. A total of 18 modules were identified, labeled by different colors. The minimum module size was set to 50 genes and the parameter mergeCutHeight was set at 0.25 to merge highly correlated modules. The intramodular connectivity (Kwithin) values were calculated as the sum of the weights of all edges for each node within a particular module. Genes displaying low network connectivity were assigned to the gray module.

To identify module-trait relationships, we calculated the Pearson correlation between the module eigengenes and sample features classified using binary or sequential values. For the tumor relationship, the cell lines 4C11− and 4C11+ were assigned to 1, and the remaining cell lines were assigned to 0 values. For the EMT relationship, the mesenchymal-like 4C and 4C11− cell lines were assigned to 1, and the remaining epithelial cell lines were assigned to 0. For the metastasis potential, replicate samples from the 4C11+ cell line were classified with the value 1 and the other cell lines 0. Lastly, for tumor progression, the melan-a, 4C, 4C11−, and 4C11+ cell line samples were classified with the sequential numbers 1, 2, 3, or 4. Module-trait associations were inferred by calculating the correlation between expression of the module eigengenes and the cell features. Only modules with a *p* value ≤ 0.1 and an absolute correlation coefficient greater than 0.3 were considered significantly correlated to a sample trait. Hubs were classified as the 10 top-ranked genes based on their Kwithin values.

The hubs from the modules that showed positive correlation with tumor progression (red, blue, light cyan, and pink), EMT (red, salmon, magenta, and turquoise), and metastasis (midnight blue, green yellow, yellow, blue, and light cyan) were used to build subnetworks related to these biological features. The subnetworks were built using Cytoscape version 3.8.0; only edges between hubs that had weights larger than 0.2 were considered. Edge weights were determined as the correlation coefficients of gene pairs raised by the soft threshold of 10. Nodes were colored according to their module assignments, and node sizes were adjusted based on their degree in each subnetwork. Only the largest subgraph was considered in each case.

### Analysis of the prognostic value of transcriptional modules and hubs using human melanoma data

The connection between co-expression modules and prognostic genes was explored using gene expression and clinical data from the TCGA (Cancer Genome Atlas [78]) and the Leeds Melanoma Project melanoma cohorts [79]. Selected genes were mapped to their human orthologs via BioMart [80] and used for further analysis. Hypergeometric tests were performed to determine whether the modules were enriched in either (i) the 500 top-ranked prognostic genes (by *p* value) from the TCGA SKCM data or (ii) the significant prognostic genes (*p* < 0.05) identified in the multivariate analyses of the Leeds Melanoma Cohort data. Modules with a *p* value < 0.05 were considered significantly enriched with prognostic genes.

First, the enrichment of prognostic genes in the modules was assessed using publicly available clinical and gene expression skin cutaneous melanoma (SKCM) data (Cancer Genome Atlas [78]) from The Cancer Genome Atlas (TCGA) and the GEPIA2 software [33]. The SKCM cohort includes 458 patients (99 primary and 359 metastatic tumors). Kaplan-Meier curves were built using overall survival as the clinical outcome and separating samples based on the median values for each gene (high and low expression groups). GEPIA2 uses the log-rank test for hypothesis evaluation; Cox proportional hazard ratios and 95% confidence intervals were included in the survival plots. The prognostic value of the hubs was assessed individually or using the 10-hub signature in each module. The multi-gene signature analysis was performed using the mean expression value of the genes as the signature score [33].

Second, the overrepresentation of prognostic genes in specific modules was evaluated using transcriptomic and clinical data from the Leeds Melanoma Cohort ([79, 81]; European Genome-Phenome Archive accession number EGAS00001002922). This is a population-based sample of primary melanoma patients with a median follow-up time of 8 years. Multivariate survival analyses (MA) were performed by dividing patients into “high” and “low” groups based on the median expression of each gene and evaluating prognostic significance by Cox proportional hazards regression. Age at diagnosis, sex, and tumor site were adjusted (MA1 analysis) before further including AJCC stage (8th edition) and mitotic rate as covariates (MA2). The statistical analysis was conducted in STATA v14 (Stata Corp, Texas, USA).

### Functional and histone mark enrichment analyses of genes in each module

Functional enrichment analysis was performed on the gene subsets in each module using the software Enrichr [82]. Biological terms from the Gene Ontology (GO), KEGG, Reactome, WikiPathways, and BioCarta databases were considered. The data from the ENCODE Histone Modifications database [83] was also used to agnostically identify histone marks that could be related to the gene co-expression signatures. Only functions and histone marks with multiple-testing adjusted *p* values lower than 0.05 were considered significant. Hypergeometric tests were also carried out to assess the enrichment of genes involved in histone modification in the modules, using information from the GO term “histone modification” (*n* = 440 genes, GO 0016570). Hierarchical clustering analyses were performed with the gplots package in R, using the (i) histone modification genes included in the co-expression modules or the data from single or combinatorial histone marks, (ii) the Pearson correlation as the distance metric, and the (iii) complete linkage clustering method.

### Gene expression analysis by quantitative reverse transcription PCR

The gene expression of histone-modifying enzymes previously involved in tumor progression [84] was also investigated. The transcriptional levels of histone acetyltransferases (*Hat1*, *Ep300*, *Kat2a*, and *Kat5*), deacetylases (*Hdac1*, *Hdac3*, *Hdac6*, and *Hdac11*), methyltransferases (*Nsd1* and *Setd2*), or demethylases (*Kdm2a* and *Kdm2b*) were assessed by RT-qPCR. .One microgram of total RNA was used for cDNA synthesis using the QuantiTect Reverse Transcription Kit following the manufacturer’s instructions. The data was normalized using the β-actin gene as an endogenous control. Statistical analyses were made by comparing the expression of each gene across the cell lines using one-way ANOVA and the Tukey’s multiple comparison test. Genes were considered differentially expressed when the multiplicity adjusted *p* values were lower than 0.05.

The primer sequences used were as follows: *Nsd1* F: 5′-GTTGCCAATAGGAGGCCATA-3′ and R: 5′-GTCTGCCTTCAAACATGACG-3′; *Setd2* F: 5′-TTGTGGTTTGGCCGCCTT-3′ and R: 5′- GAGGGAGCACACATTCCAAGTT-3′; *Hdac1* F: 5′-TGAGGAGGACCCTGACAAAC-3′ and R: 5′-GTTCTTGCGACCACCTTCTC-3′, *Ep300* F: 5′-CTTCAGACAAGTCTTGGCATAGT-3′ and R: 5′-CCGACTCCCATGTTGAGGTTT-3′; *Hdac3* F: 5′ ACCGTGGCGTATTTCTACGAC-3′ and R: 5′-CAGGCGATGAGGTTTCATTGG-3′; *Hdac6* F: 5′-TCCACCGGCCAAGATTCTTC-3′ and R: 5′-CAGCACACTTCTTTCCACCAC-3′; *Hdac11* F: 5′-GTGTACTCACCACGTTACAACA-3′ and R: 5′-GCTCGTTGAGATAGCGCCTC-3′; *Kdm2a* F: 5′-AACACACAGAAGGGGATTGAAAT-3′ and R: 5′- GCACCATATTCTCAAGCCTGG-3′; *Hat1* F: 5′-ACACCAACACAGCAATCGAG-3′ and R: 5′-TGTAACCGAAAGCAGTTTCATCA-3′; *Kat2a* F: 5′-AACCTGAGCGAGTTGTGCC-3′ and R: 5′-GCCGGTTAATCTCGTCCTCTG-3′; *Kat5* F: 5′-AGCGGAAATCTAATTGCTTGGG-3′ and R: 5′ TTCGTGGTGCTGACGGTATTC 3′; *Kdm2b* F: 5′-AGGCAAGTTTAACCTCATGCTC-3′ and R: 5′- ACACCCTCCGATTCCTTAATCTT-3′; *Actb* F: 5′-ACCGTGAAAAGATGACCCAG-3′ and R: 5′-GTACGACCAGAGGCATACAG-3′.

### Global profiling of histone modifications by mass spectrometry

#### i) Histone extraction, propionylation, and digestion

The histone protein extracts were obtained as described by Sidoli et al. [85]. The cell pellets were suspended in 1 ml of a nuclear isolation buffer (NIB) (15 mM Tris, 60 mM KCl, NaCl 15 mM, 15 mM NaCl, 5 mM MgCl_2_, 1 mM CaCl_2_, 250 mM sucrose supplemented with phosphatase inhibitor cocktails (Sigma), and 1 mM DTT, 10 μM sodium butyrate) and centrifuged at 700×*g* for 5 min. The supernatant was removed, and the cell pellet was lysed by resuspension in 1 ml of NIB containing 0.2% NP-40 supplemented with protease inhibitors, homogenized by pipetting and incubated for 10 min on ice. The lysates were centrifuged at 1000×*g* for 10 min at 4 °C. The pellet was washed twice with NIB buffer. The cell nuclei were resuspended in 500 μL of 0.2 M H_2_SO_4_ and incubated under constant agitation for 2 h at 4 °C. Supernatants were collected after centrifugation at 3400×*g* at 4 °C for 5 min, and histones were precipitated with 33% of trichloroacetic acid (TCA) overnight at 4 °C. The pellet was washed twice with ice-cold acetone. Histone peptides were subjected to chemical derivatization using propionic anhydride and trypsin digestion. Twenty micrograms of dried histones were dissolved with 20 μl of 50 mM NH_4_HCO3, pH 8.0. Ten microliter of the propionylation reagent (1:3, v:v, propionic anhydride:propanol) was added to each sample, followed by incubation at 37 °C for 15 min. To ensure complete histone propionylation, samples were subjected to a second round of propionylation. The histones were resuspended in 50 mM NH_4_HCO_3_ at 1 μg/μl and incubated with trypsin (Promega) (1:20) (w:w) at 37 °C for 6 h. A third round of propionylation was performed to chemically modify the N-terminus of digested peptides. Finally, samples were desalted using C18 Zip-tips (Merck).

#### ii) Liquid chromatography-mass spectrometry (LC-MS/MS)

The peptides (~ 3 μg per run) were suspended in 0.1% formic acid and fractionated in a reverse phase capillary column (10 cm × 75 μm, packed with 5 μm of C18 Aqua resin-Phenomenex) coupled to a nanoHPLC (Thermo) inline to a LTQ-Orbitrap Velos (Thermo Scientific). Fractionation was performed under a 60-min gradient: 0–28% buffer B (0.1% formic acid in acetonitrile) in 45 min, 28–80% in 13 min; 80–5% in 2 min at a flow rate of 300 nL min−1. The MS voltage was 1.9 kV, and the capillary temperature was set at 200 °C. The equipment was operated in data independent acquisition (DIA) with 50 m/z to select the precursor ions for fragmentation as described in [86]. For each cycle, one full MS1 was followed by 8 MS2 scans using an isolation width of 50 m/z starting from 325 to 1075 m/z.

#### iii) Analysis of histone modification data

Data was processed by the EpiProfile program [87] using *Mus musculus* as a database entry. The relative abundance of each histone PTM was calculated as the area of a particular peptide divided by the total area for that peptide in all modified forms. The PTM levels were statistically compared between the cell lines using one-way ANOVA followed by the Tukey’s multiple comparison test, using GraphPad Prism 8.0 (GraphPad Software, Inc., San Diego, CA). Only histone PTMs with multiplicity adjusted *p* values lower than 0.05 were considered significant. C-fuzzy means clustering analysis was performed using the online tool available at http://computproteomics.bmb.sdu.dk/Apps/FuzzyClust/. The fuzzifier value and the number of clusters were automatically estimated by the algorithm. All raw files were deposited at PRIDE number PXD019313. The association of the histone marks with the biological features was based on their relative abundance in the cell lines. Histone marks that were statistically altered in 4C cells compared to melan-a or displayed two statistically significant changes in the comparisons between two consecutive cell lines in the model were related to tumor progression. Histone PTMs with similar expression in the mesenchymal versus epithelial cells were linked to EMT, and histone PTMs showing statistically significant changes between the 4C11− and 4C11+ cell lines were related to melanoma metastasis.

## Supplementary information

**Additional file 1: Figure S1.** Scatter plots showing the relationship between the soft threshold power β (x-axis) and the scale-free topology model fit (R^2^) or mean connectivity values (y-axis). A β value equal to 10 was chosen, resulting in an R^2^ higher than 0.8 and a mean connectivity still above 0.

**Additional file 2: Figure S2.** Module assignment using the weighted gene co-expression network analysis (WGCNA) algorithm. (A) Cluster dendrogram and module assignment for each gene. The topological overlap dissimilarity measure was used in the average linkage hierarchical clustering and module assignments are shown labeled by different colors. Eighteen modules were identified by analyzing the 5000 most variable genes. The minimum module size was set to 50 genes. (B) Clustering trees showing the similarity between the modules calculated based on the distance between their module eigengenes.

**Additional file 3: Figure S3.** Pearson correlation of the histone peptides’ abundance ratios among replicates. The relative abundance levels of 245 histone PTM peptides (containing single or multiple PTM combinations) were determined in biological triplicates for each cell line. The Pearson correlation coefficients are reported in each pairwise comparison and the strength of the correlation is shown by the intensity of the red color. Overall, the 4C11+ cell triplicates (11+ rep1, rep2 and rep3) were less correlated with the triplicates from the other cell lines.

**Additional file 4: **Supplementary Tables. **Table S1.** Abundance of histone PTM (ratio values) single marks obtained from the EpiProfile output. **Table S2.** Abundance of histone PTM (ratio values) combinatory marks obtained from the EpiProfile output. **Table S3.** c-fuzzy means clustering of the combinatorial histone modifications. **Table S4.** Multiplicity adjusted p values from the one-way ANOVA followed by Tukey's post hoc tests. Pairwise statistical comparisons were performed using relative abundance levels of each single post translational modification (PTM) from the histones 3 and 4. **Table S5.** Summary of one-way ANOVA results obtained for all histone PTM single marks. Only PTMs that are differentially expressed in at least one pairwise comparison are depicted.

## Data Availability

The RNA-seq data are accessible through the gene expression Omnibus (https://www.ncbi.nlm.nih.gov/geo/) under the accession number GSE149884. Histone modifications raw files are available at PRIDE number PXD019313.

## Abbreviations

AJCC: American Joint Committee on Cancer
EMT: Epithelial-to-mesenchymal transition
FDR: False discovery rate
GO: Gene Ontology
HAT: Histone acetyltransferase
HDAC: Histone deacetylase
HR: Hazard ratio
KDM: Lysine demethylase
KMT: Lysine methyltransferase
Kwithin: Intramodular connectivity
MA: Multivariate survival analysis
qPCR: Quantitative real time PCR
PTMs: Post-translational modifications
SKCM: Skin cell melanoma
TCGA: The Cancer Genome Atlas
WGCNA: Weighted gene co-expression network analysis
