# Supplementary figures and images for: Gene co-expression and histone modification signatures are associated with melanoma progression, epithelial-to-mesenchymal transition, and metastasis
Source: Clin Epigenetics. 2020 Aug 24;12:127. doi: 10.1186/s13148-020-00910-9 (PMC7444266; doi:10.1186/s13148-020-00910-9)

Scale independence

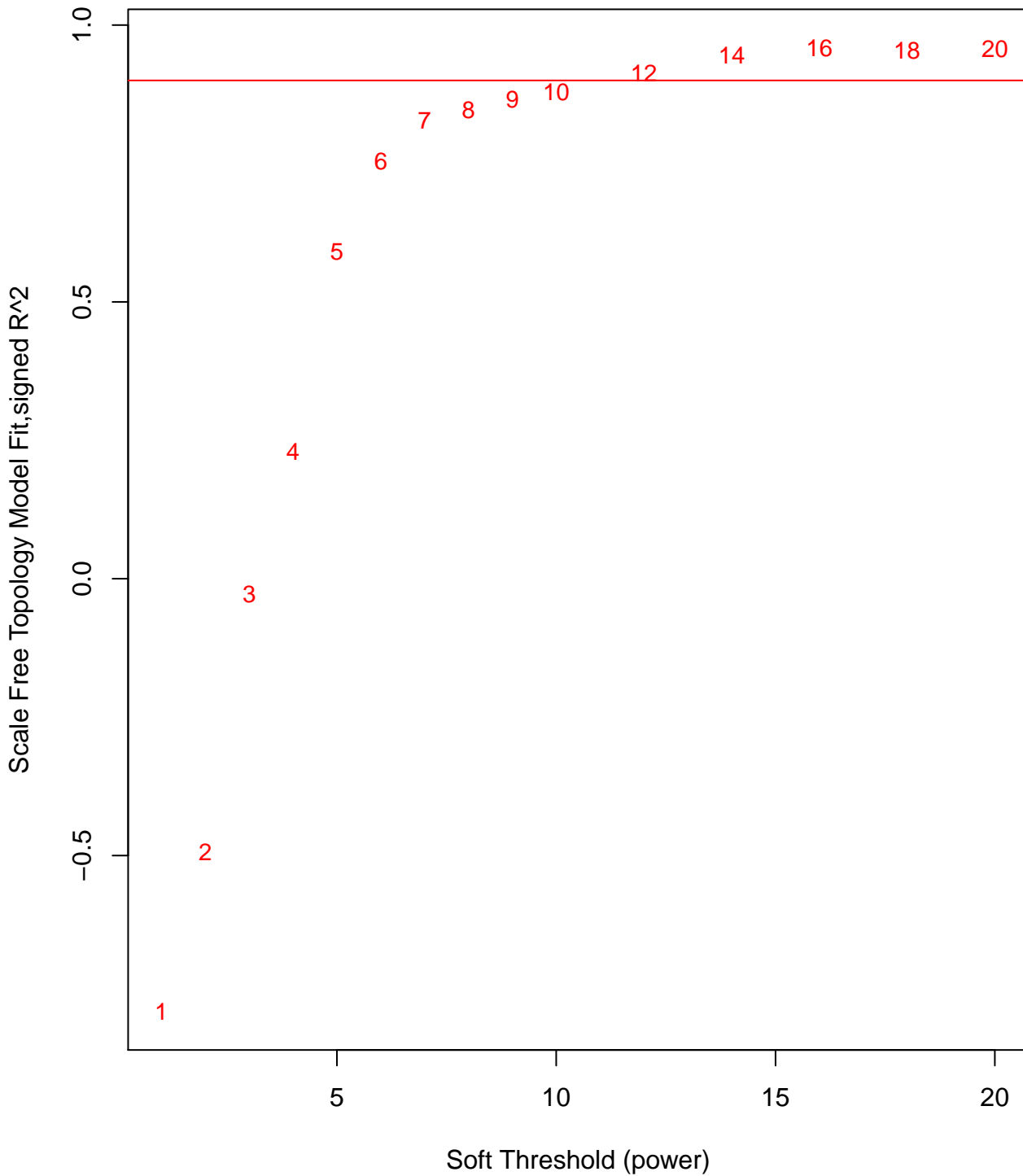

Mean connectivity

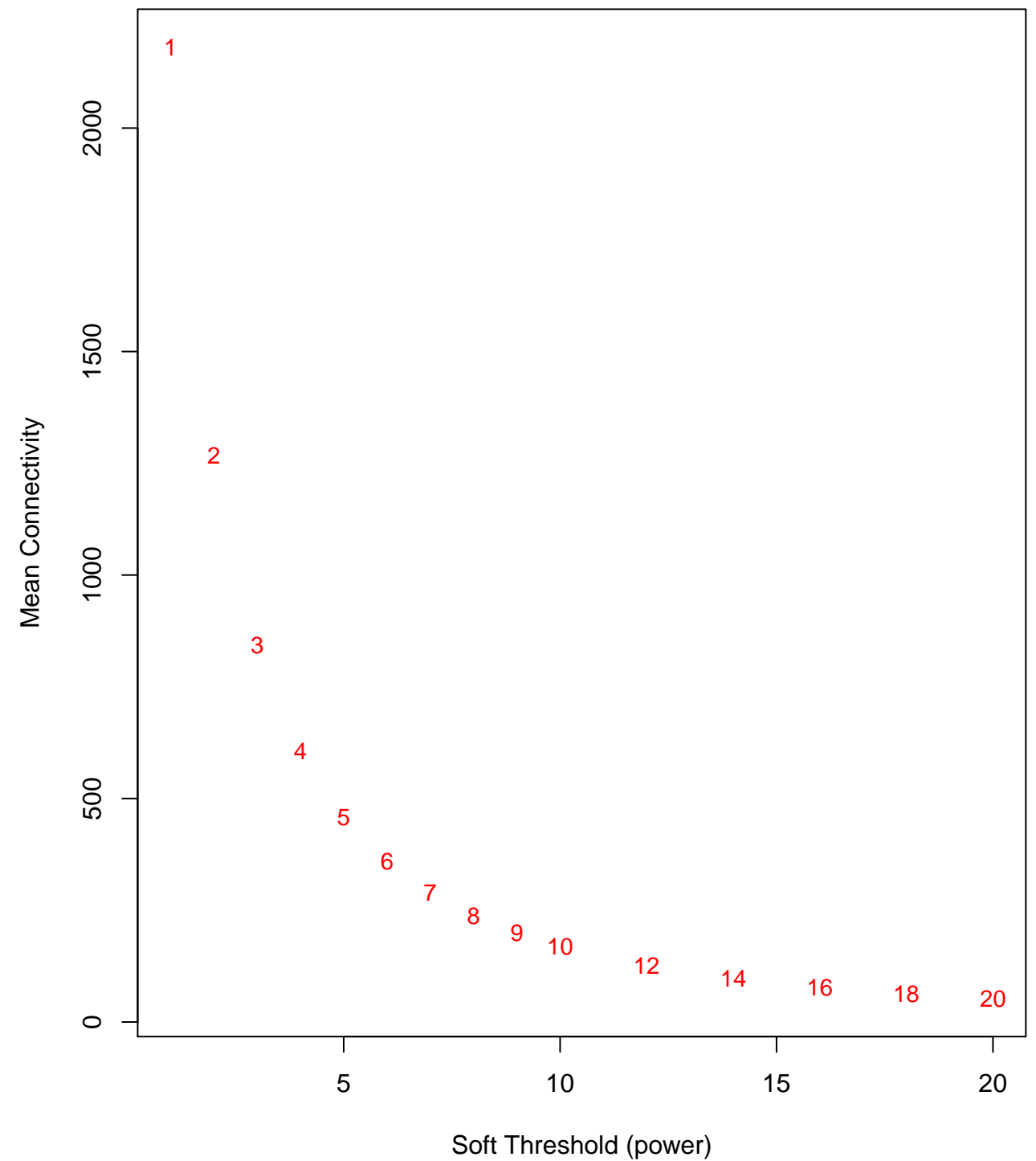

Supplement: Supplementary file 1 — Additional file 1: Figure S1. Scatter plots showing the relationship between the soft threshold power β (x-axis) and the scale-free topology model fit (R2) or mean connectivity values (y-axis). A β value equal to 10 was chosen, resulting in an R2 higher than 0.8 and a mean connectivity still above 0. [file 13148_2020_910_MOESM1_ESM.pdf]

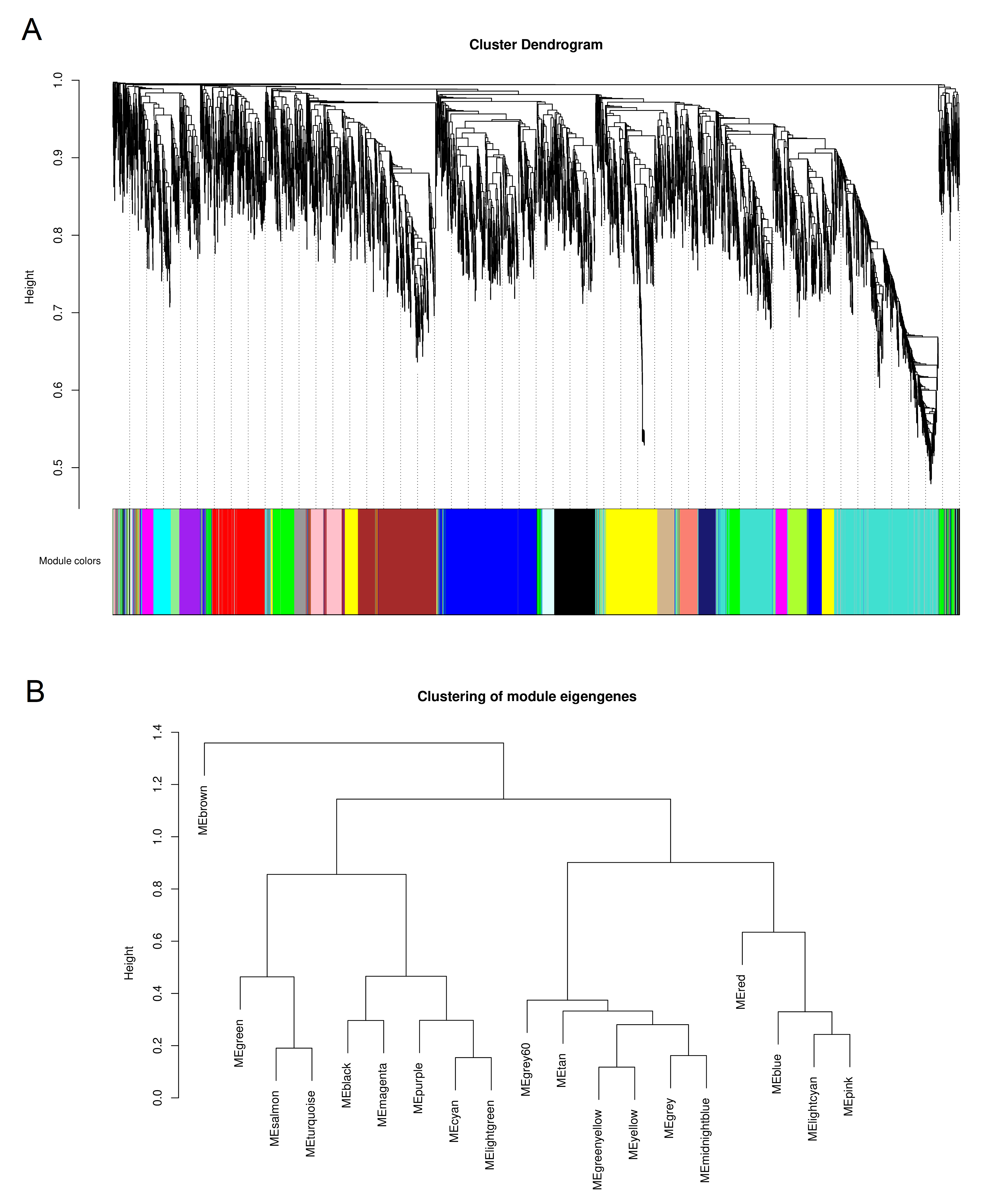

Supplement: Supplementary file 2 — Additional file 2: Figure S2. Module assignment using the weighted gene co-expression network analysis (WGCNA) algorithm. (A) Cluster dendrogram and module assignment for each gene. The topological overlap dissimilarity measure was used in the average linkage hierarchical clustering and module assignments are shown labeled by different colors. Eighteen modules were identified by analyzing the 5000 most variable genes. The minimum module size was set to 50 genes. (B) Clustering trees showing the similarity between the modules calculated based on the distance between their module eigengenes. [file 13148_2020_910_MOESM2_ESM.tif]

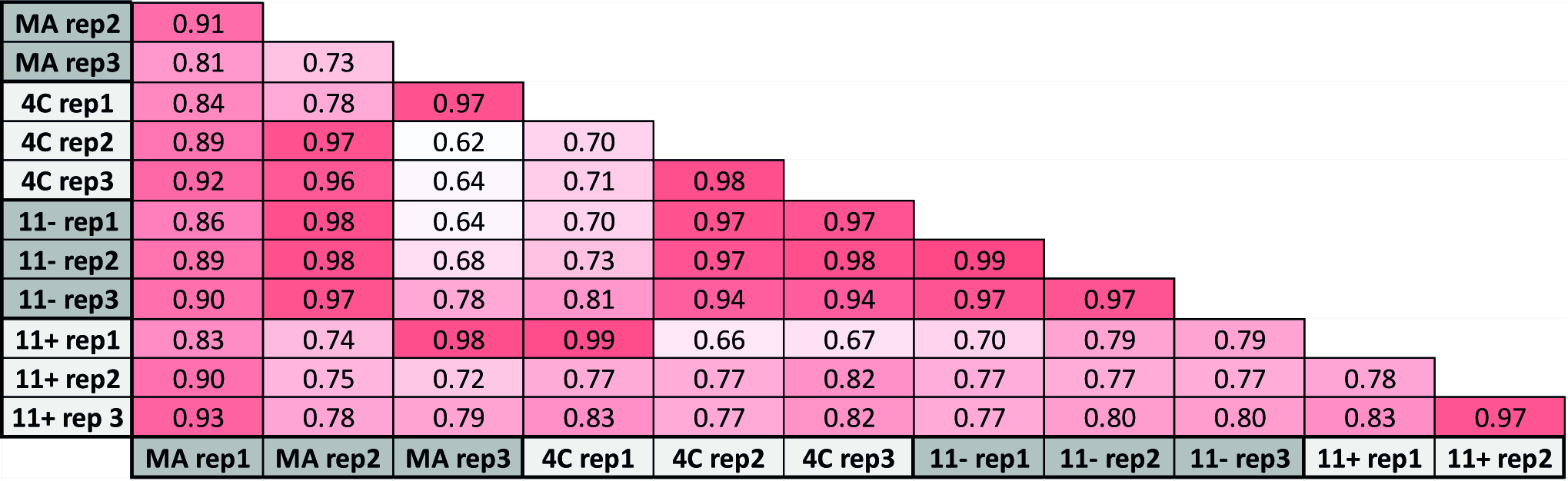

Supplement: Supplementary file 3 — Additional file 3: Figure S3. Pearson correlation of the histone peptides’ abundance ratios among replicates. The relative abundance levels of 245 histone PTM peptides (containing single or multiple PTM combinations) were determined in biological triplicates for each cell line. The Pearson correlation coefficients are reported in each pairwise comparison and the strength of the correlation is shown by the intensity of the red color. Overall, the 4C11+ cell triplicates (11+ rep1, rep2 and rep3) were less correlated with the triplicates from the other cell lines. [file 13148_2020_910_MOESM3_ESM.tif]
